# Fabrication of γ-Fe_2_O_3_ Nanowires from Abundant and Low-cost Fe Plate for Highly Effective Electrocatalytic Water Splitting

**DOI:** 10.1038/s41598-020-62259-6

**Published:** 2020-03-25

**Authors:** Sivaranjani Arumugam, Yuhki Toku, Yang Ju

**Affiliations:** Department of Micro-Nano Mechanical Science and Engineering, Graduate School of Engineering, Nagoya University, Nagoya, 464-8603 Japan

**Keywords:** Nanoscience and technology, Nanoscale materials, Nanowires

## Abstract

Water splitting is thermodynamically uphill reaction, hence it cannot occur easily, and also highly complicated and challenging reaction in chemistry. In electrocatalytic water splitting, the combination of oxygen and hydrogen evolution reactions produces highly clean and sustainable hydrogen energy and which attracts research communities. Also, fabrication of highly active and low cost materials for water splitting is a major challenge. Therefore, in the present study, γ-Fe_2_O_3_ nanowires were fabricated from highly available and cost-effective iron plate without any chemical modifications/doping onto the surface of the working electrode with high current density. The fabricated nanowires achieved the current density of 10 mA/cm^2^ at 1.88 V vs. RHE with the scan rate of 50 mV/sec. Stability measurements of the fabricated Fe_2_O_3_ nanowires were monitored up to 3275 sec with the current density of 9.6 mA/cm^2^ at a constant potential of 1.7 V vs. RHE and scan rate of 50 mV/sec.

## Introduction

Renewable energies such as solar, water and wind energies are clean and highly abundant resources which are not depleted by use. Hence, the utility of these resources has received greater concern among researchers. Particularly, the selection of best technology to utilize water/solar energy is an important issue. Among various methods (i) photoelectrochemical (PEC) and (ii) electrochemical water splitting (conventionally known as water electrolysis) are prominent methods to directly utilize renewable energy (water/solar) into chemical energy for the production of hydrogen and oxygen^1^. Compared with fossil fuels hydrogen has three to four fold higher energy density as well as it never pollutes the environment by producing CO_2_ gas, hence, PEC and electrochemical water splitting received greater consideration^2–5^. Usually, in PEC water splitting, semiconductor materials have been used as photoelectrode. These semiconductors consisting of two energy bands viz., valence and conduction band, and the energy difference between these two bands are known as band gap, the band gap range for semiconductor materials are 1 to 5 eV. The theoretical band energy level for water splitting is 1.23 eV. But in practical, the band gap of the semiconductor should be greater than 1.23 eV to avoid thermodynamic losses (~0.4 eV) and over potentials (~0.3 eV). Therefore, the band gap of semiconductor should be ~1.9 eV for effective water splitting^6^. Based on these backgrounds, thin film metal oxides such as TiO_2_, ZnO, Fe_2_O_3_, Cu_2_O and WO_3_ have already been used extensively as a semiconductor material for PEC water splitting. For example, ZnO/ZnFe_2_O_4_ core–shell heterojunction photoelectrode was prepared and the PEC performance of ZnO/ZnFe_2_O_4_ was optimized by depositing NiOOH^7^. Also, one dimensional (1D) Fe_2_O_3_ nanorod arrays with dual-axial gradient doping of Zr and Sn facilitate the electron-carrier concentration and charge-separation efficiency across the dual-axial direction of Fe_2_O_3_ nanorods^8^. To enhance the light harvesting and charge separation nature of Cu_2_O, the spatially separated noble-metal cocatalysts viz., Au and Pt nanolayers have deposited onto Cu_2_O nanogranules^9^. Recently, 1D/0D WO_3_/CdS heterojunction photoanodes was fabricated and modified with dual co-catalysts viz., NiOOH and Co-Pi to enhance the water splitting efficiency, where the heterojunctions prohibit the recombination of photo-generated electron-hole pairs and CdS enhances the absorption of the light^10^. Moreover, the fabrication of polycrystalline α-Fe_2_O_3_ nanowire array through oxidation-assisted stress-induced atomic diffusion under water vapor environment and also single-crystal α-Fe_2_O_3_ nanowire array by stress-induced atomic diffusion with surface polishing treatment have been reported^11,12^. However, the low energy conversion efficiency of PEC water splitting restricts its large scale application.

In contrast, electrochemical water splitting method has high efficiency, excellent adaptability and flexibility, which can efficiently produce hydrogen with high purity. In electrochemical water splitting, anodic oxygen evolution reaction (OER) and cathodic hydrogen evolution reaction (HER) are two critical half-cell reactions. The standard oxidation potential for OER is 1.23 V vs. RHE and standard reduction potential for HER is 0 V vs RHE for electrochemical water splitting. But in practice, it should be larger than 1.23 V to avoid some unfavorable factors such as activation energy, ion and gas diffusion, electrolyte concentration, wire and electrode resistances, electrolyte diffusion blockage, bubble formation and thermodynamic losses, which leads additional potential over the standard potential^13^. Initially, anodic and cathodic reactions were catalyzed by Ru, Ir and their oxides catalysts^14,15^. However, these noble metals cannot be used for commercial applications due to their high cost and lack of abundance. At this situation transition metals such as Ni, Co, Fe, Mn, W, etc. and their oxides have been extensively used as electrocatalytic materials for effective electrochemical water splitting^16,17^. Among them iron oxides received greater attention to split water efficiently. In addition, it is highly abundant, non-toxic to the environment, cost effective and also stable in aqueous solution^18,19^. Moreover, the demerit like slow reaction kinetics can be solved by fabricating an electrode with nanostructured morphology. Based on this phenomenon wide varieties of nanostructures have been reported so far.

Recently, Sharifi *et al*.^20^ reported the current density of γ-Fe_2_O_3_ modified carbon nanotubes as 1 mA/cm^2^ at 1.57 V vs RHE in 0.1 M KOH and Tavakkoli *et al*.^21^ reported the current density of γ-Fe_2_O_3_ nanoparticle decorated carbon nanotubes as 10 mA/cm^2^ at the potential of 1.61 and 1.57 V vs. RHE in 0.1 and 1 M NaOH, respectively. Moreover, Ashwani Kumar *et al*.^22^ reported the current density of NiFe-NC obtained from the composite of NiO and α/γ-Fe_2_O_3_ as 10 mA/cm^2^ at the potential of 1.67 V vs. RHE in 1 M KOH. Recently, Davodi *et al*.^23^ reported that MWNTs functionalized with nitrogen-rich emeraldine salt (ES-MWNT) as a promising catalyst support to boost the electrocatalytic activity of magnetic maghemite (γ-Fe_2_O_3_) NPs and the current density of the electrocatalyst (Ni@γ-Fe_2_O_3_/ES-MWNT) was measured as 10 mA/cm^2^ at the potential of 1.49 V vs RHE in 1 M NaOH. In addition, Chandrasekaran *et al*.^24^ employed γ-Fe_2_O_3_ with reduced graphene oxide for PEC water splitting and the photocurrent density of the RGO/γ-Fe_2_O_3_ nanocomposite was reported as 6.74 mA/cm^2^ at 1.80 V vs RHE in 1 M NaOH. Even though these reports are available for electrocatalytic water splitting, still it is challenging to attain greater efficiency at low applied potential through highly available and cost-effective materials.

Usually, highly available and low-cost materials are sluggish in nature which needs some modifications, but the materials and methods used to modify the electrodes are highly expensive. Hence, we intended to use a pure, low cost and highly obtainable material without any additional modification with greater efficiency even at very low applied potential. Moreover, it has been reported that facile thermal oxidation is a simple and effective method to grow nanostructured materials including iron oxides^25–27^. Therefore, in the present study, we fabricated γ-Fe_2_O_3_ nanowire array from low cost and easily available Fe plate by adopting simple thermal treatment. The formation of Fe_2_O_3_ nanowires and its structural morphology was investigated by microscopic and spectroscopic techniques viz., Field emission scanning electron microscopy (FESEM), Energy-dispersive X-ray spectroscopy (EDS), Fourier-transform infrared spectroscopy (FTIR), X-ray diffraction (XRD), Transmission electron microscopy (TEM), Selected area electron diffraction pattern (SAED) and X-ray photoelectron spectroscopy (XPS). Finally, the current density of the obtained Fe_2_O_3_ nanowires was measured by applying the materials as working electrode for electrocatalytic water splitting and the current density has reached as 10 mA/cm^2^ at 1.88 V vs. RHE. The value obtained from bare Fe_2_O_3_ is comparable with previous results, where chemical modifications or doping is necessary^20–24^.

## Results and Discussion

### Morphologies and structural characterizations

After thermal treatment, the morphology of Fe plate with scratched surface was compared with unscratched Fe plate using FESEM analysis (Fig. S1, Supporting Information). From the images it is observed that the scratched surface (Fig. S1(a)) showed substantial growth of nanowires than unscratched surface (Fig. S1(b)) and the corresponding mechanisms (Fig. S2) for nanowire formation has drawn on the basis of experimental results and previous reports^11,12^. A reducing environment or straining the surface by applying external force has already been employed to prepare γ-Fe_2_O_3_^28–30^. Here, the surface of the Fe plates has been strained by uniform scratching, as a result, the effect of strained surface generates applied stresses. This applied stresses fasten the grain boundaries efficiently from the very beginning of the surface oxidation process. The strained surface encourages the initial compressive stress on the surface of the Fe plate, which clogged the volume expansion of oxide layer. Consequently, the vertical stress gradient occurred and expedites the iron ion diffusion and followed to improve the nanowire growth. Moreover, the strained surface can enhance the surface roughness of the Fe plate and thus expand the volume of Fe_2_O_3_ layer. The enhanced volume expansion of Fe_2_O_3_ layer can increase the tensile strength of the Fe plate and thus facilitates the driving force of iron ion diffusion. In unscratched Fe_2_O_3_, grain boundaries have been created by the effect of internal stresses at a respective temperature. The obtained grain boundaries provides route to iron ion diffusion. However, the inadequate internal stress generated in unscratched Fe_2_O_3_ produces lesser grain boundaries for iron ion diffusion and thus indicates fewer growths of nanowires in Figs. S1(b) and S2(b).

**Figure 1:**
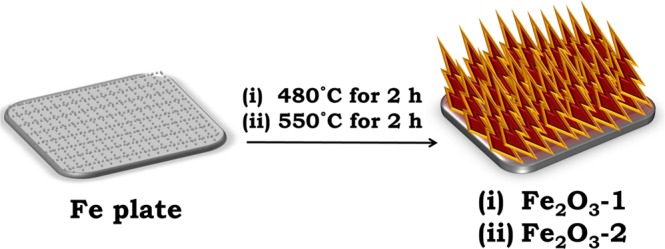
Schematic diagram for fabrication of Fe_2_O_3_ nanowires.

**Figure 2:**
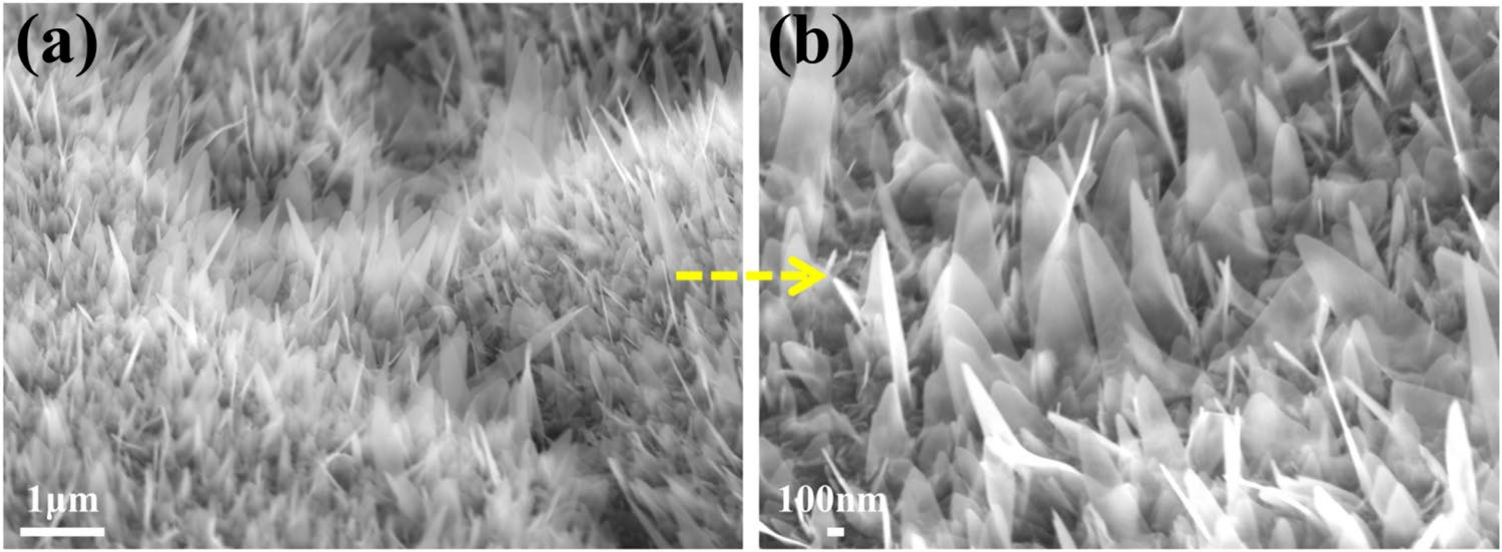
FESEM images of Fe_2_O_3_-1 nanowires at (**a**) low and (**b**) high magnification.

**Figure 3:**
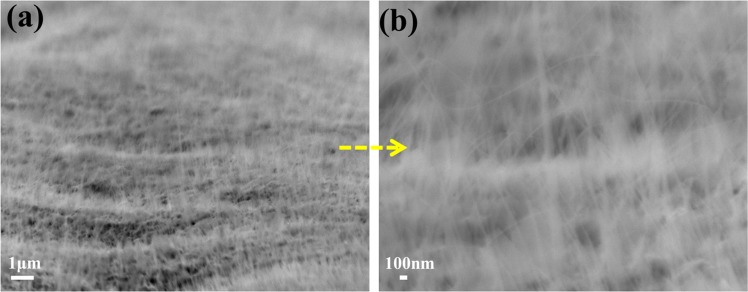
FESEM images of Fe_2_O_3_-2 nanowires at (**a**) low and (**b**) high magnification.

The schematic diagram for nanowire formation is shown in Fig. 1. The element distributions of the fabricated Fe_2_O_3_ electrodes were quantitatively measured by EDS analysis. The top view FESEM image of Fe_2_O_3_-1 and the corresponding EDS mapping of Fe_2_O_3_, Fe and O are shown in Fig. S3, Supporting Information. Moreover, the corresponding EDS spectrum is shown in Fig. S4, Supporting Information and the weight percentage of Fe and O elements in Fe_2_O_3_-1 were found as 71.14 and 28.86%, respectively. Similarly, the EDS mapping of Fe_2_O_3_, Fe and O in Fe_2_O_3_-2, and the corresponding EDS spectrum are shown in Figs. S5 and S6, Supporting Information, the weight percentage of Fe and O elements in Fe_2_O_3_-2 were found as 70.02 and 29.98%, respectively. Therefore, the mapping images obtained from Fe_2_O_3_-1 and Fe_2_O_3_-2 clearly indicates the abundant and uniform distributions of Fe and O elements in Fe_2_O_3_ layer and the appropriate weight percentage of Fe and O are further evidence for the formation of Fe_2_O_3_. The side view of nanowires growth was captured from low to high magnifications as shown in Figs. 2, 3 and S7, and the corresponding cross-section FESEM images are shown in Fig. S8. In Fig. 2, the obtained images of Fe_2_O_3_-1 showed highly dense and ordered growth of Fe_2_O_3_ nanowires on iron plate than Fe_2_O_3_-2. The specific reason for the growth of nanowire is atomic diffusion of Fe atom due to the existence of compressive stress. Also, the continuous supply of Fe and O at higher temperature for prolonged duration enhances the growth of nanowires^31,32^.

In briefly, the as-received Fe-sheet was subjected to pretreatment using distilled water and ethanol and then individually employed for annealing process at 480 and 550 °C for 2 h and thus obtained Fe_2_O_3_-1 and Fe_2_O_3_-2, respectively. The thickness of the as-prepared samples viz., Fe_2_O_3_-1 and Fe_2_O_3_-2 was measured by FESEM analysis. Moreover, to measure the thickness of each layer obtained after thermal treatment, the side view of the cross-sectional FESEM images were taken by cutting the samples as shown in Fig. S9, Supporting Information. The average thickness of Fe_2_O_3_ and Fe layers in Fe_2_O_3_-1 were measured as 3.61 and 90.76 μm, respectively. Similarly, in Fe_2_O_3_-2, the average thickness of Fe_2_O_3_ and Fe layers were measured as 8.32 and 82.84 μm, respectively. On comparing the results, the thickness of Fe_2_O_3_ layer increased on increasing the temperature and the remained Fe layer decreased due to the promoted oxidation. Moreover, the cross-sectional EDS image of Fe_2_O_3_-1 and the elemental mapping of Fe and O are shown in Fig. S10, Supporting Information. The obtained results supported the uniform distribution of elements with two different layers such as Fe_2_O_3_ and Fe which indicates the formation of Fe_2_O_3_ nanowires onto Fe plate. Similar observation was also noticed for Fe_2_O_3_-2 as shown in Fig. S11, Supporting Information. Then, the phase transitions of Fe_2_O_3_ nanostructures were investigated by FTIR analysis. Fig. S12 (Supporting Information) shows the characteristic peaks for γ-Fe_2_O_3_ at 440, 558 and 636 cm^−1^, α-Fe_2_O_3_ at 471 cm^−1^ and Fe_3_O_4_ at 565 cm^−1^ which indicates the existence of different phase in the obtained materials. Papadas *et al*.^33^ reported the similar peaks for γ-Fe_2_O_3_ at 442, 558 and 636 cm^−1^, α- Fe_2_O_3_ at 471 cm^−1^ and Fe_3_O_4_ at 565 cm^−1^. Zhang *et al*.^34^ also reported the same observations for γ, α-Fe_2_O_3_ and Fe_3_O_4_.

**Figure 4:**
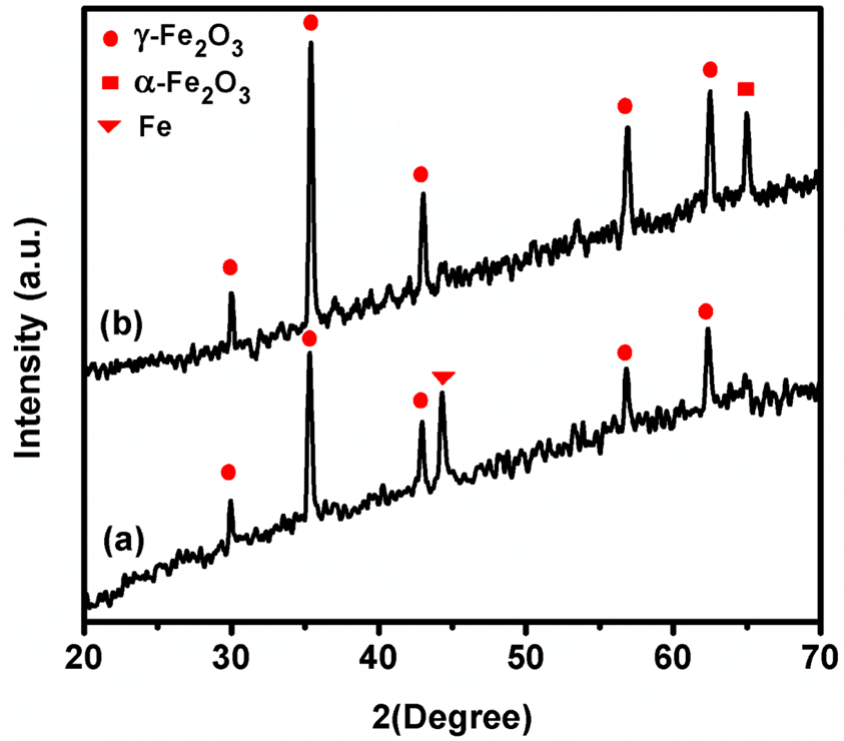
XRD patterns of (**a**) Fe_2_O_3_-1 and (**b**) Fe_2_O_3_-2 nanowires.

The crystalline properties and crystalline phase of the obtained Fe_2_O_3_ was investigated by XRD analysis. In Fig. 4(a), Fe_2_O_3_-1 showed 2θ peaks at 29.9°, 35.3°, 42.9°, 56.8° and 62.3° which are relevant to (220), (311), (400), (511) and (440) crystal planes of γ-Fe_2_O_3_, respectively. Similarly, in Fig. 4(b), Fe_2_O_3_-2 showed the peaks at 30.0°, 35.4°, 43.0°, 56.9° and 62.5° due to (220), (311), (400), (511) and (440) crystal planes of γ-Fe_2_O_3_, respectively. In addition, the peak observed at 44.3° for the crystal plane of (110) in Fe_2_O_3_-1 belongs to iron plate. In Fe_2_O_3_-2, the new peak observed at 65.0° was relevant to the crystal plane of (300) of α-Fe_2_O_3_. These peaks and its planes are agreed well with the standard XRD database of γ- and α- Fe_2_O_3_ (JCPDS no: 39-1356 and 33-0664)^34^. Hence, the disappearance of Fe peak and the appearance of new peak for α-phase clearly indicate the phase transition of iron oxide (γ to α) from lower temperature to higher temperature. Usually, Fe has various oxidation states but the common oxidation state of Fe is +2 and +3. Initially Fe donate 2e^−^ to oxygen to attain Fe^2+^ oxidation state and which produce FeO, when increasing the temperature and duration, FeO replaced by Fe_3_O_4_ in such case the oxidation state of Fe in Fe_3_O_4_ (Magnetite) is +2 and +3, and it is ferrimagnetic in nature, further heating leads β/γ- Fe_2_O_3_, but β and γ phases are intermediate phases. Since β-phase is highly unstable, not easy to separate, but there is some possibility of getting γ-Fe_2_O_3_ (Maghemite), and it is also ferrimagnetic in nature but the oxidation state is +3. At higher temperature, γ-phase is obviously replaced by the stable α-phase (hematite). The phase transition temperature can vary from materials to materials based on the thickness and purity of the Fe-precursor which have been chosen for analysis and the equipment (oven/furnace/ceramic heater/CVD) used for annealing process.

**Figure 5:**
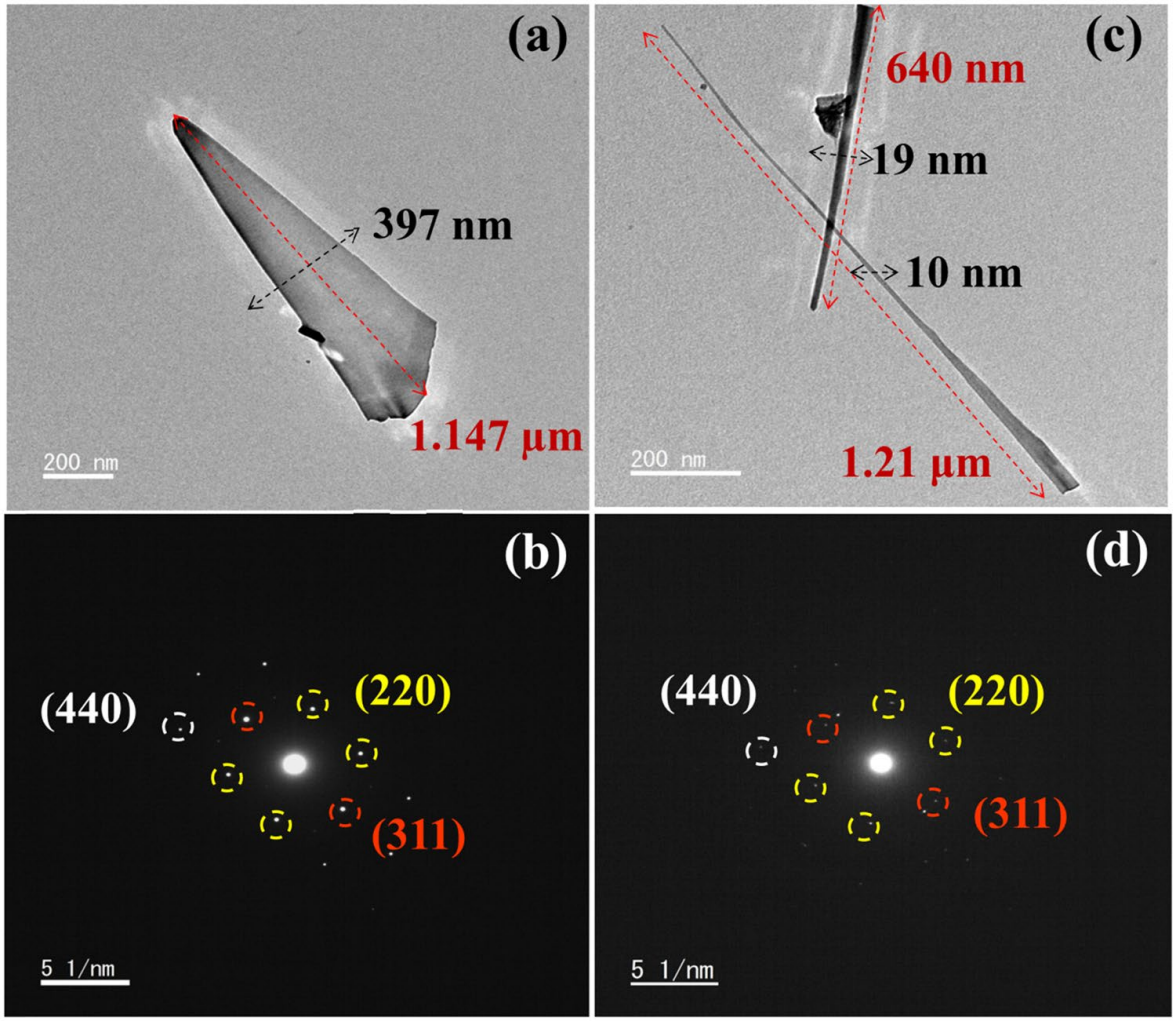
TEM images and SAED patterns of Fe_2_O_3_-1 (**a,b**) and Fe_2_O_3_-2 (**c,d**).

The morphology, size and shape of single nanowire were investigated by TEM analysis. As shown in Fig. 5(a,c) the morphology of the nanowires were found smooth. The diameter and length of Fe_2_O_3_-1 was measured as 397 nm and 1.14 µm (Fig. 5(a)). Whereas, the TEM image of Fe_2_O_3_-2 exhibit different sized nanowires, which are due to the fact that not all nanowires were nucleated at the same time. In Fig. 5(c), the diameter and length of the longer one was found as 10 nm and 1.21 µm, respectively, and the shorter one showed the diameter and length of 19 and 640 nm, respectively. It is suggest that the thinner nanowires are longer than thicker one at a specific oxidation temperature and time^35^. On comparing the TEM results of Fe_2_O_3_-1 and Fe_2_O_3_-2, the nanowires found in Fe_2_O_3_-2 was thinner and longer than Fe_2_O_3_-1 nanowire. The reason is considered to be due to the increased compressive stress with the increase of temperature from 480 to 550 °C which influenced the growth of longer nanowires observed in Fe_2_O_3_-2 than that of Fe_2_O_3_-1^31,32^. The crystal planes and crystalline structure of single Fe_2_O_3_ nanowire was further confirmed by SAED pattern. As shown in Fig. 5(b,d), the diffraction spots obtained from Fe_2_O_3_-1 and Fe_2_O_3_-2 were indexed as (220), (311) and (440) planes by measuring the distance of the spots using its appropriate d-spacing, which are corresponding to the cubic crystal cells of γ-Fe_2_O_3_. Further, the obtained results clearly indicate that the fabricated γ-Fe_2_O_3_ nanowires were existed in single crystalline structure and the results are agreed well with previous reports^36,37^.

**Figure 6:**
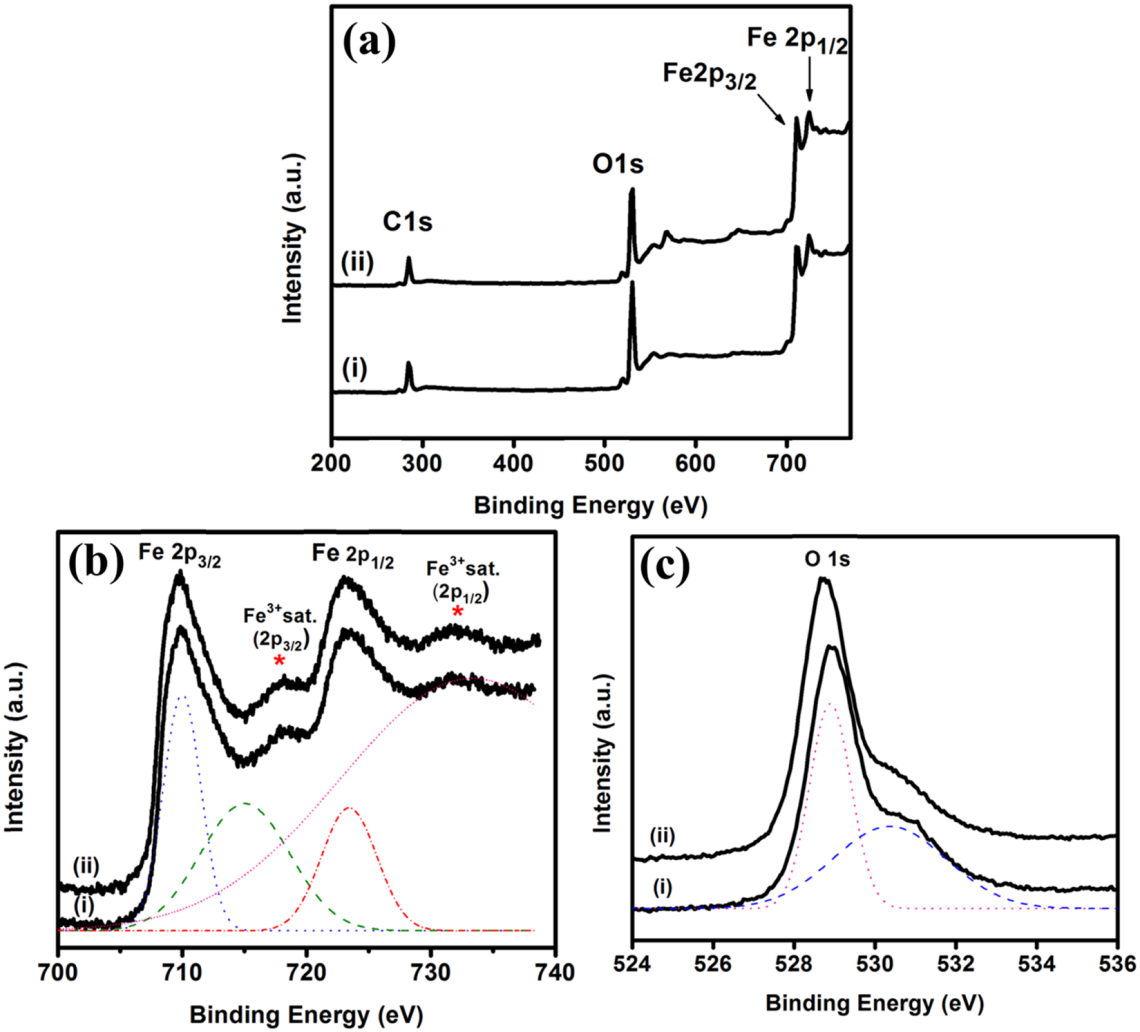
(**a**) XPS survey spectra of (i) Fe_2_O_3_-1 and (ii) Fe_2_O_3_-2. Deconvoluted XPS spectra of (**b**) Fe 2p and (**c**) O 1s of (i) Fe_2_O_3_-1 and (ii) Fe_2_O_3_-2.

The biding energy, oxidation state and phase transition of Fe_2_O_3_-1 and 2 were further verified by XPS analysis. In Fig. 6(a), the survey spectra of Fe_2_O_3_-1 and Fe_2_O_3_-2 showed the characteristic peaks at 530.3, 710.3 and 724.3 eV corresponding to O 1s, Fe 2p_3/2_ and Fe 2p_1/2_, respectively. The deconvoluted XPS spectra of both Fe_2_O_3_-1 and 2 showed the major peaks for Fe 2p_3/2_ at 709.9 eV and Fe 2p_1/2_ at 723.3 eV (Fig. 6(b)). Further, the charge transfer satellite peak for Fe 2p_3/2_ and Fe 2p_1/2_ was observed at 717.8 eV and 731.8 eV which are strong evidence for the oxidation state of Fe^3+^ in γ-Fe_2_O_3_. This observation was agreed well with previous literatures^38,39^. Also, Wang *et al*.^40^ and Pereira *et al*.^41^ reported similar binding energies for Fe 2p and the charge transfer satellite peaks for +3 oxidation state of γ-Fe_2_O_3_ phase. The deconvoluted peaks for O 1s observed at 529.0 eV indicates the biding energy of lattice oxygen in Fe_2_O_3_ and a shoulder at 530.8 eV indicates the presence of chemisorbed oxygen (OH^−^) species on sample surface (Fig. 6(c)). This interference was matched with the reported results^35,42,43^. On comparing the results, the nanowires obtained from Fe_2_O_3_-1 and Fe_2_O_3_-2 showed similar characteristic peaks in XPS spectra which indicate that the two samples were exist in same form and also in same phase^35^.

### Electrochemical measurements

**Figure 7:**
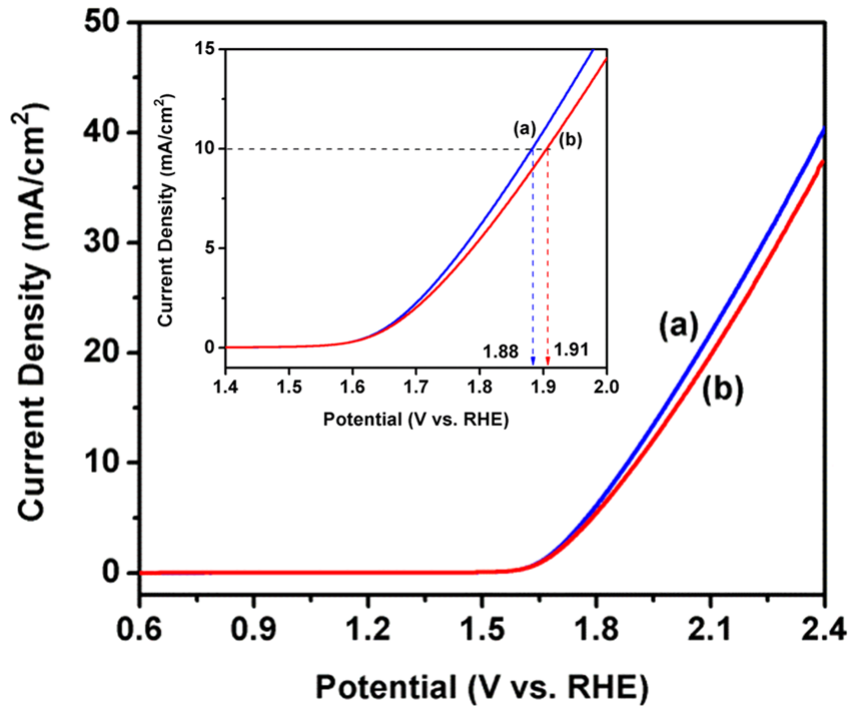
LSV measurements of (**a**) Fe_2_O_3_-1 and (**b**) Fe_2_O_3_-2 (inset shows the potentials of (**a**) Fe_2_O_3_-1 and (**b**) Fe_2_O_3_-2 at 10 mA/cm^2^).

The electrochemical water splitting measurements were performed in 1 M NaOH electrolyte solution at pH 13.5 with the help of conventional three electrode system. The fabricated Fe_2_O_3_-1 and Fe_2_O_3_-2 nanowires were applied directly as working electrode without any chemical modification or doping, which is highly advantageous for large scale application in near future and also it can reduce the cost of expensive chemical substances/dopants to modify the electrode surface. As shown in Fig. 7, the linear sweep voltammograms (LSV) of Fe_2_O_3_-1 and Fe_2_O_3_-2 illustrate the enriched current density of 10 mA/cm^2^ at the potentials of 1.88 and 1.91 V vs. RHE with 50 mV/sec scan rate. Even though the applied potential is higher than previous results obtained from chemically modified or doped γ-Fe_2_O_3_ nanostructures, no one has reported improved performance for bare Fe_2_O_3_ electrode (Table 1)^20–24^. Specifically, three important factors such as, (i) highly dense and ordered growth of nanowires, (ii) more abundant of γ-phase and (iii) single crystalline structure played essential role to improve the performance of the as-prepared Fe_2_O_3_ electrodes. In detail, the highly dense and ordered growth of Fe_2_O_3_ nanowires (obtained after scratching the electrode surface, Figs. S1(a) and S2(a), Supporting Information) produced high surface area. The more abundant γ-phase on Fe_2_O_3_ electrode acts as an efficient electrocatalyst for oxygen evolution reaction due to its greater electrical conductivity for rapid electron transfer. Also, γ-Fe_2_O_3_ provided substantial and synergetic effect on improving catalysis by producing more catalytic active sites. Therefore, the enormous surface area and the enhanced electrocatalytic active sites offered more capability to adsorb OH^−^ ions (presented in the electrolyte solution) which leads the enhancement in catalysis and fast electron transfer towards oxygen evolution reaction, as a result of the improved performance in Fe_2_O_3_ electrode (Fig. 7). Moreover, the sharp edges of the Fe_2_O_3_ nanowires highly supported to the charge transfer process of electrode/electrolyte interface i.e. electrons obtained from oxygen evolution reaction were easily moved from electrolyte solution to Fe_2_O_3_ electrode. In addition, the single crystalline structure of Fe_2_O_3_ electrode (Fig. 5) played vital role to enhance the electron movements from Fe_2_O_3_ to Fe layer and which also improved the performance of the as-prepared Fe_2_O_3_ electrode.

**Table 1:** Comparison of current density of recently reported electrode materials.

Electrolyte	Semiconductors (bare and modified)	Photocurrent density (mA/cm^2^)	Potential (V)	Reference
0.1 M KOH	MR@NCNTs/CP	1	1.57 V vs RHE	^20^
0.1 M NaOH	γ-Fe_2_O_3_/CNT	10	1.61 V vs RHE	^21^
1 M NaOH			1.57 V vs RHE	
1 M KOH	NiFe-NC from NiO and α/γ-Fe_2_O	10	1.67 V vs RHE	^22^
1 M NaOH	Ni@γ-Fe_2_O_3_/ES-MWNT	10	1.49 V vs RHE	^23^
1 M NaOH	RGO/γ-Fe_2_O_3_	6.74	1.80 V vs RHE	^24^
1 M NaOH	γ-Fe_2_O_3_ NWs	10	1.88 V vs RHE	Present Work

**Figure 8:**
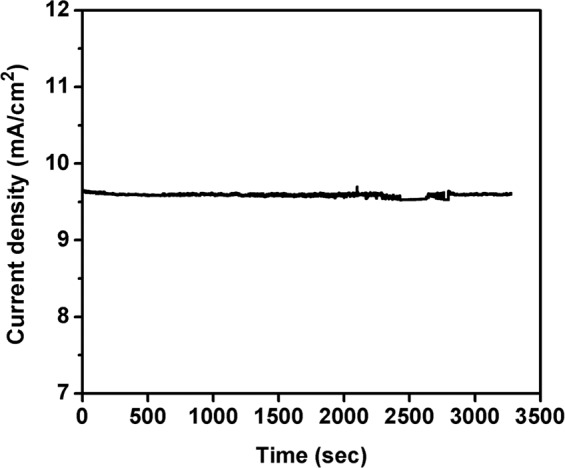
Stability measurement of Fe_2_O_3_-1 at 1.7 V vs. RHE for 3275 sec at 50 mV/sec scan rate.

**Figure 9:**
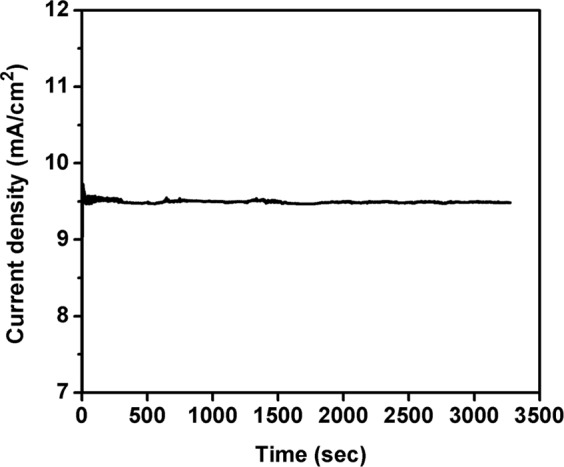
Stability measurement of Fe_2_O_3_-2 at 1.7 V vs. RHE for 3275 sec at 50 mV/sec scan rate.

On comparing the results obtained from electrochemical measurements, a small potential difference has been noticed between Fe_2_O_3_-1 and Fe_2_O_3_-2 (Fig. 7). This is due to non-uniform and lack of nanowires growth, and increased thickness of Fe_2_O_3_ layer found in Fe_2_O_3_-2. The non-uniform and lack of nanowires growth slightly reduced the surface area and catalytic active sites of the electrode, and the thickness slowdown the electron movement from Fe_2_O_3_ to Fe layer during water splitting. Therefore, Fe_2_O_3_-2 electrode showed slight increment in applied potentials than Fe_2_O_3_-1. Moreover, stability measurements were performed for both Fe_2_O_3_-1 and Fe_2_O_3_-2 against current density vs. time at a constant potential of 1.7 V vs. RHE with the scan rate of 50 mV/sec as shown in Figs. 8 and 9. From the result it is observed that there is no substantial change in stability of the electrode up to 3275 sec and the current density was observed as 9.6 mA/cm^2^ for Fe_2_O_3_-1 and 9.5 mA/cm^2^ for Fe_2_O_3_-2, respectively. The vigorous formation of bubbles during stability measurement (see Supplementary Movie 1) is directly corresponding to the oxygen and hydrogen evolution reactions on both anode and cathode surfaces, respectively^44,45^, and the observation was explained by the following possible reaction mechanism^13^. The OER reaction on γ-Fe_2_O_3_ was also supported by previous report^21^. Specifically, catalytic reactions involved in four steps such as (a) adsorption of reactant molecules onto catalyst surface, (b) diffusion of reactants onto catalyst surface, (c) reaction takes place from reactants and which produce products, and finally (d) desorption of products from catalyst surface. The electrocatalytic measurement of the present investigation has been performed under alkaline condition, therefore the OER reaction proceeds by the following reaction steps:

1. Adsorption of ‘OH^−^’ (present in alkaline electrolyte solution) onto γ-Fe_2_O_3_ catalyst surface.
  i $${{\rm{OH}}}^{-}+{{\rm{M}}}^{\ast }\to {{\rm{M}}}^{\ast }-{\rm{OH}}+{{\rm{e}}}^{-}$$
2. Where, M*- represents the active sites of γ-Fe_2_O_3_ metal catalyst. The obtained M*-OH was further reacted with OH^−^ and formed M*-O.
  ii $${{\rm{M}}}^{\ast }-{\rm{OH}}+{{\rm{OH}}}^{-}\to {{\rm{M}}}^{\ast }-{\rm{O}}+{{\rm{H}}}_{2}{\rm{O}}+{{\rm{e}}}^{-}$$
3. (The formation of O_2_ can take place in two pathways, one is direct coupling of two M*-O, but the thermodynamic barrier of this reaction is high.
  iii $${{\rm{M}}}^{\ast }-{\rm{O}}+{{\rm{M}}}^{\ast }-{\rm{O}}\to {{\rm{O}}}_{2}$$
4. In second pathway, M*-O reacts with OH^−^ and produced intermediate molecule (M*-OOH). This intermediate consequently reacted with another OH^−^ and produced O_2_.

iv $${{\rm{M}}}^{\ast }-{\rm{O}}+{{\rm{OH}}}^{-}\to {{\rm{M}}}^{\ast }-{\rm{OOH}}+{{\rm{e}}}^{-}$$

v $${{\rm{M}}}^{\ast }-{\rm{OOH}}+{{\rm{OH}}}^{-}\to {{\rm{O}}}_{2}+{{\rm{H}}}_{2}{\rm{O}}+{{\rm{e}}}^{-}$$

Moreover, it is value to mention here that *α*-Fe_2_O_3_ (hematite) offers a favorable combination of good visible light absorption up to 590 nm hence it has been widely used as photoactive material for solar water splitting^46^, but recently few reports are demonstrated the excellent electrocatalytic activity of chemically modified γ-Fe_2_O_3_ in electrocatalytic water splitting^20–24^. The morphology of γ-Fe_2_O_3_ electrode used for stability measurements was observed by FESEM analysis and the images are shown in Fig. S16 (Supporting Information). This image shows that there was no substantial change in the electrode morphology and the result reveals the best stability of γ-Fe_2_O_3_ electrode in alkaline solution and the resistivity against corrosion. The samples after stability measurements were also measured by XPS analysis and the results are shown in Fig. S17 (Supporting Information). These results suggested that γ-Fe_2_O_3_ NWs are highly efficient material to achieve excellent current density and stability. Moreover, the single crystalline structure of γ-Fe_2_O_3_ is also an important factor to increase the efficiency of electrocatalytic water splitting, however, the preparation of γ-Fe_2_O_3_ with single crystalline structure is still a challenging so far^36,37^.

**Figure 10:**
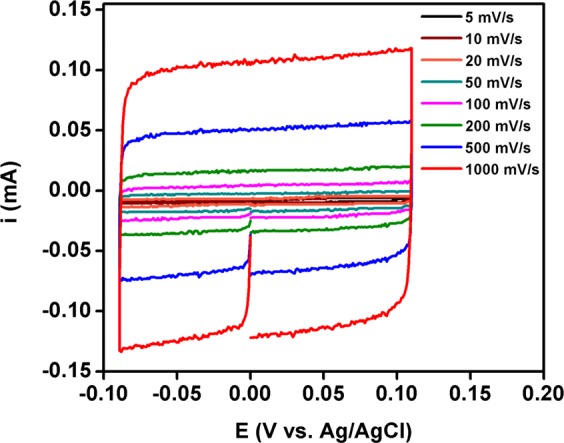
Cyclic voltammogram measured for Fe_2_O_3_-1 at non-faradaic region (−0.090 V to 0.110 V vs. Ag/AgCl) with different scan rates (5, 10, 20, 50, 100, 200, 500 and 1000 mV/sec).

**Figure 11:**
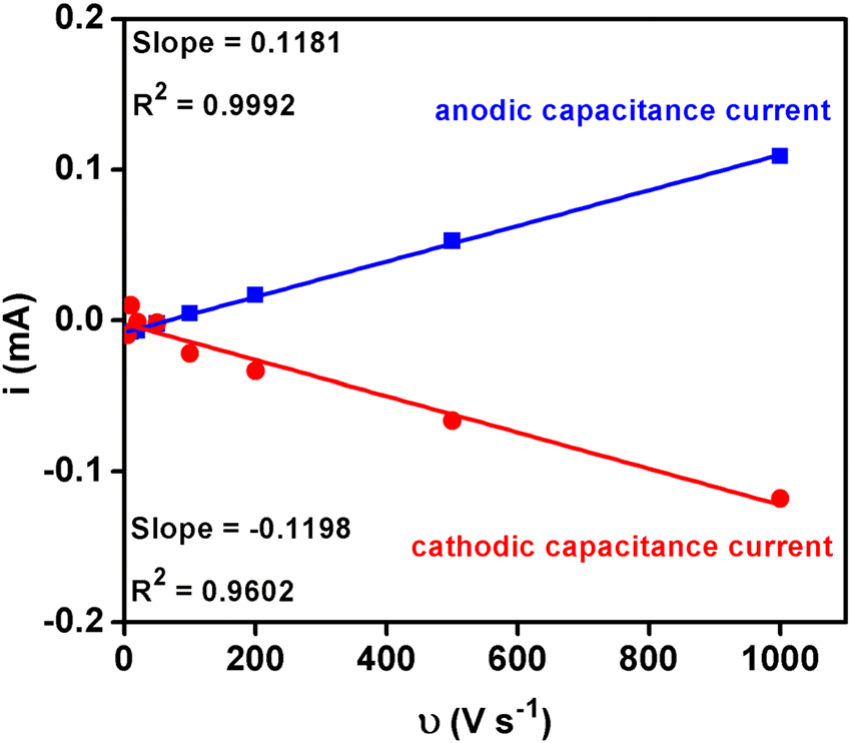
Anodic and cathodic capacitance currents measured for Fe_2_O_3_-1 at 0.026 V vs. Ag/AgCl and plotted as a function of scan rate.

In addition, the electrochemical capacitances have been calculated through double layer capacitance of the fabricated electrode material in 1 M NaOH^22,47,48^. To measure double layer capacitance, cyclic voltammetry measurements were performed at non-faradaic region (i.e., −0.090 to 0.110 V vs. Ag/AgCl is converted as 0.904 to 1.104 V vs. RHE) with different scan rates viz., 5, 10, 20, 50, 100, 200, 500 and 1000 mV/sec as shown in Fig. 10. All measured current at this non-faradaic region was assumed to as double-layer charging current. Then the cathodic and anodic capacitance current was measured at 0.026 V vs Ag/AgCl (i.e. 1.020 V vs. RHE) from each scan rate. The obtained capacitance currents were plotted against scan rates (Fig. 11). Then the double-layer capacitance was calculated from the average absolute value of the both cathodic and anodic slope of the linear fitting of the plot, i.e. double layer capacitance (C_DL_) was measured from the slope of charging currents (i_c_) as a function of scan rate (υ) as shown in Eq. 1.

1 $${{\rm{i}}}_{{\rm{c}}}=\upsilon {{\rm{C}}}_{{\rm{DL}}}$$

The electrochemical double-layer capacitance measured from the scan-rate study for the Fe_2_O_3_-1 catalyst was C_DL_ = 0.119 mF. The electrochemically active surface area (ECAS) of Fe_2_O_3_-1 was measured from the obtained electrochemical double-layer capacitance of the catalytic surface by using Eq. 2.

2 $${\rm{ECAS}}={{\rm{C}}}_{{\rm{DL}}}/{{\rm{C}}}_{{\rm{s}}}$$

Where C_s_ is the specific capacitance of the sample or the capacitance of a planar surface per unit area under identical electrolyte conditions. The average specific capacitance of 1 M NaOH was reported as 0.040 mF cm^−2 ^^21,47,48^. Then, the electrochemically active surface area of Fe_2_O_3_-1 was measured as ECAS = 3 cm^2^ by applying the C_s_ value of 1 M NaOH in Eq. 2. Further, the roughness factor (RF) of the Fe_2_O_3_-1 electrode has been calculated by using Eq. 3.

3 $${\rm{RF}}={\rm{ECSA}}/{\rm{GSA}}$$

where GSA is geometric surface area, the geometric surface area of the catalytic material used in this study was 0.25 cm^2^. Then, the roughness factor (RF) of the working electrode Fe_2_O_3_-1 was measured as RF = 12. Therefore, it has been cleared that the measured electrochemically active surface area (3 cm^2^) and surface roughness (12) of Fe_2_O_3_-1 played vital role to contribute to the high activity of the fabricated material. The higher activity of the fabricated Fe_2_O_3_ electrocatalyst was corresponding to the existence of γ-phase in Fe_2_O_3_-1. Tavakkoli *et al*.^21^ also reported that γ-Fe_2_O_3_ nanoparticles showed higher activity than α-Fe_2_O_3_.

## Conclusions

In conclusion, we have successfully fabricated highly effective, easily available and low-cost γ-Fe_2_O_3_ nanowires by adopting a simple thermal oxidation method. The current density of the newly fabricated nanowires array was measured as 10 mA/cm^2^ at 1.88 V vs. RHE with the scan rate of 50 mV/sec in 1 M NaOH solution and the stability of the working electrode were maintained up to 3275 sec and the current density was observed as 9.6 mA/cm^2^. This result is in a similar or higher order with that of other reported chemically modified/doped γ-Fe_2_O_3_ nanostructured electrodes. The unique electrocatalytic nature, γ-phase, high density and single crystalline structure of the fabricated γ-Fe_2_O_3_ nanowires played a vital role to obtain higher current density and long durability. The obtained simple bare electrode can definitely possess cost effectiveness, high availability, higher energy conversion, compatibility and activity than existing modified electrodes. Hence, the newly fabricated Fe_2_O_3_ nanowires obtained without using any co-catalyst, doping agents or any other modification onto electrode materials are expected to be a prominent candidate for electrochemical water splitting in near future compared with existing modified/doped nanostructured materials.

## Methods

The schematic representation for the fabrication of γ-Fe_2_O_3_ was shown in Fig. 1. Initially, the commercially available Fe plate with 99.5% purity was cut into small pieces (5 mm × 10 mm), and the surface of the Fe plate (front side) which is used for electrolysis was scratched by knife. Then the plate was cleaned by dispersing with ethanol and distilled water separately and dried under room temperature. Afterwards it was introduced for thermal treatment i.e. the pretreated Fe plate was placed on ceramic boat and heated for 2 h at 480 °C by using electric furnace under air. After thermal treatment the steel blue color of the Fe plate changed into dark brown color, and which has been named as Fe_2_O_3_-1. Similarly, Fe_2_O_3_-2 has been prepared by treating the Fe plate for 2 h at 550 °C. The obtained samples were analyzed by spectroscopic and microscopic techniques. All the time three set of samples have been prepared in both experimental condition and employed for spectroscopic and microscopic analysis. The morphology and elemental mapping of the samples were monitored by Field Emission Scanning Electron Microscopy (FESEM) combined with Energy-dispersive X-ray spectroscopy (EDS) using JSM-7200F. Then the different iron oxides group present in the sample was observed by Fourier-transform infrared spectroscopy (JASCO FT/IR-4100). The crystalline properties of the Fe_2_O_3_-1 and Fe_2_O_3_-2 were investigated by X-ray diffraction analysis (XRD, ATX-G, RIGAKU with Cu Kα1). Then, the morphology, structure, size and the corresponding Selected Area Electron Diffraction pattern (SAED) of a single nanostructure was investigated by Transmission Electron Microscopy (TEM) analysis using JEM-2100. The binding affinity of iron and oxygen present in γ-Fe_2_O_3_ and the oxidation state of Fe in γ-Fe_2_O_3_ was further verified by X-ray Photoelectron Spectroscopic (XPS) analysis (ESCALAB 250). Electrochemical water splitting measurements were measured by using potentiostat (ALS/DY2325 BI-POTENTIOSTAT) in 1 M of NaOH electrolyte solution with the pH of 13.5. In this measurement, Platinum wire was used as counter electrode, Ag/AgCl as reference electrode and the fabricated Fe_2_O_3_ nanostructures (0.25 cm^2^) as working electrode. The obtained results were converted to the theoretical water splitting potential of 1.23 V vs. Reversible Hydrogen Electrode (RHE) with the help of Nernst equation.

## Supplementary information

Supplementary information.

Supplementary information2
