# Supplementary material for: Fabrication of γ-Fe2O3 Nanowires from Abundant and Low-cost Fe Plate for Highly Effective Electrocatalytic Water Splitting
Source: Sci Rep. 2020 Mar 25;10:5407. doi: 10.1038/s41598-020-62259-6 (PMC7096520; doi:10.1038/s41598-020-62259-6)
Supplement: Supplementary file 1 — Supplementary information. [file 41598_2020_62259_MOESM1_ESM.docx]

**Supplementary Information**

Fabrication of γ-Fe_2_O_3_ nanowires from abundant and low-cost Fe plate for highly effective electrocatalytic water splitting

*Sivaranjani Arumugam, Yuhki Toku and Yang Ju**

Department of Micro-Nano Mechanical Science and Engineering, Graduate School of Engineering, Nagoya University, Nagoya 464-8603, Japan
*Correspondence e-mail: ju@mech.nagoya-u.ac.jp


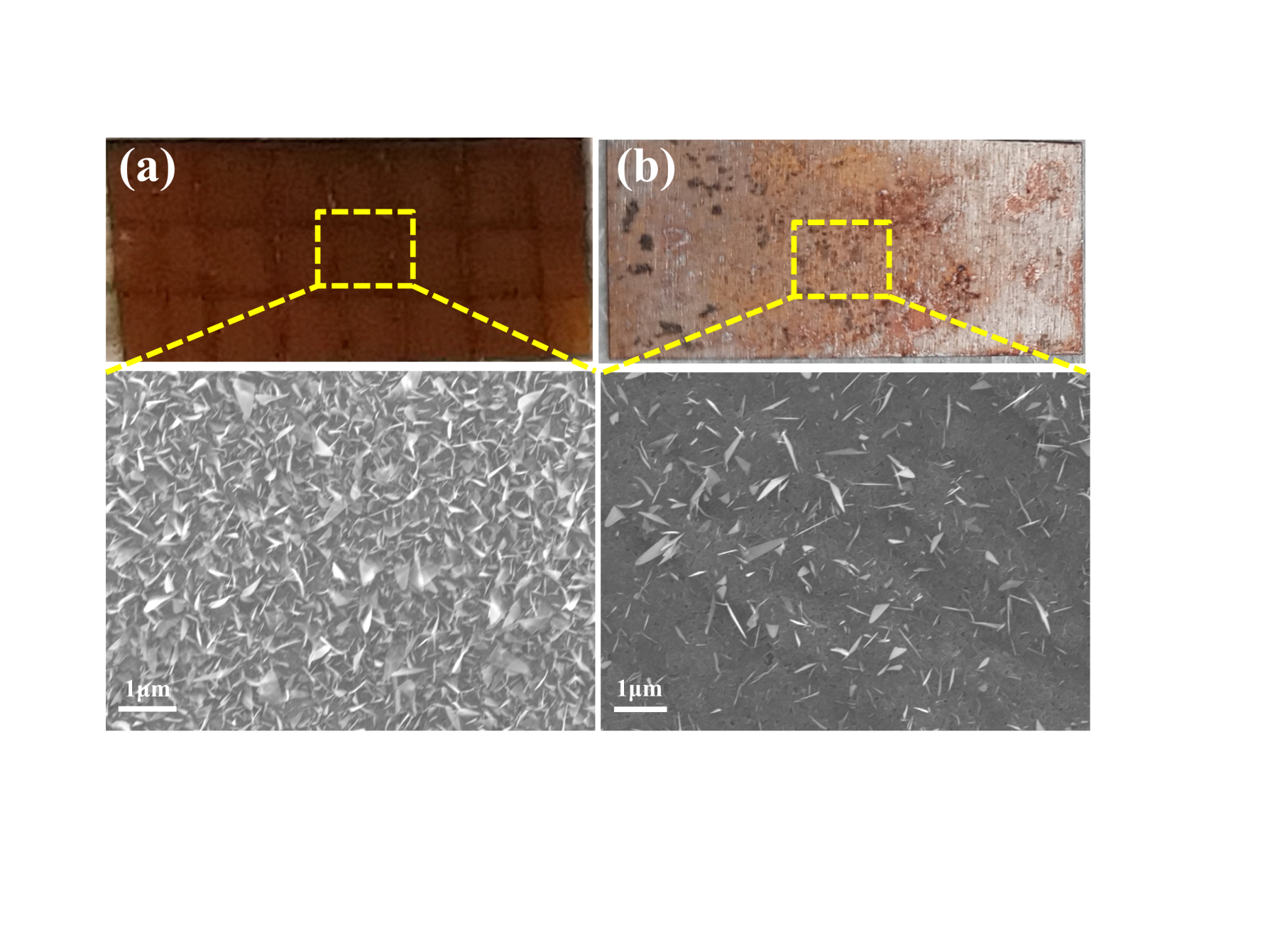


**Figure S1.** Photographs and the corresponding FESEM images (taken from top view) of
(a) surface scratched Fe plate and (b) unscratched Fe plate after thermal treatment


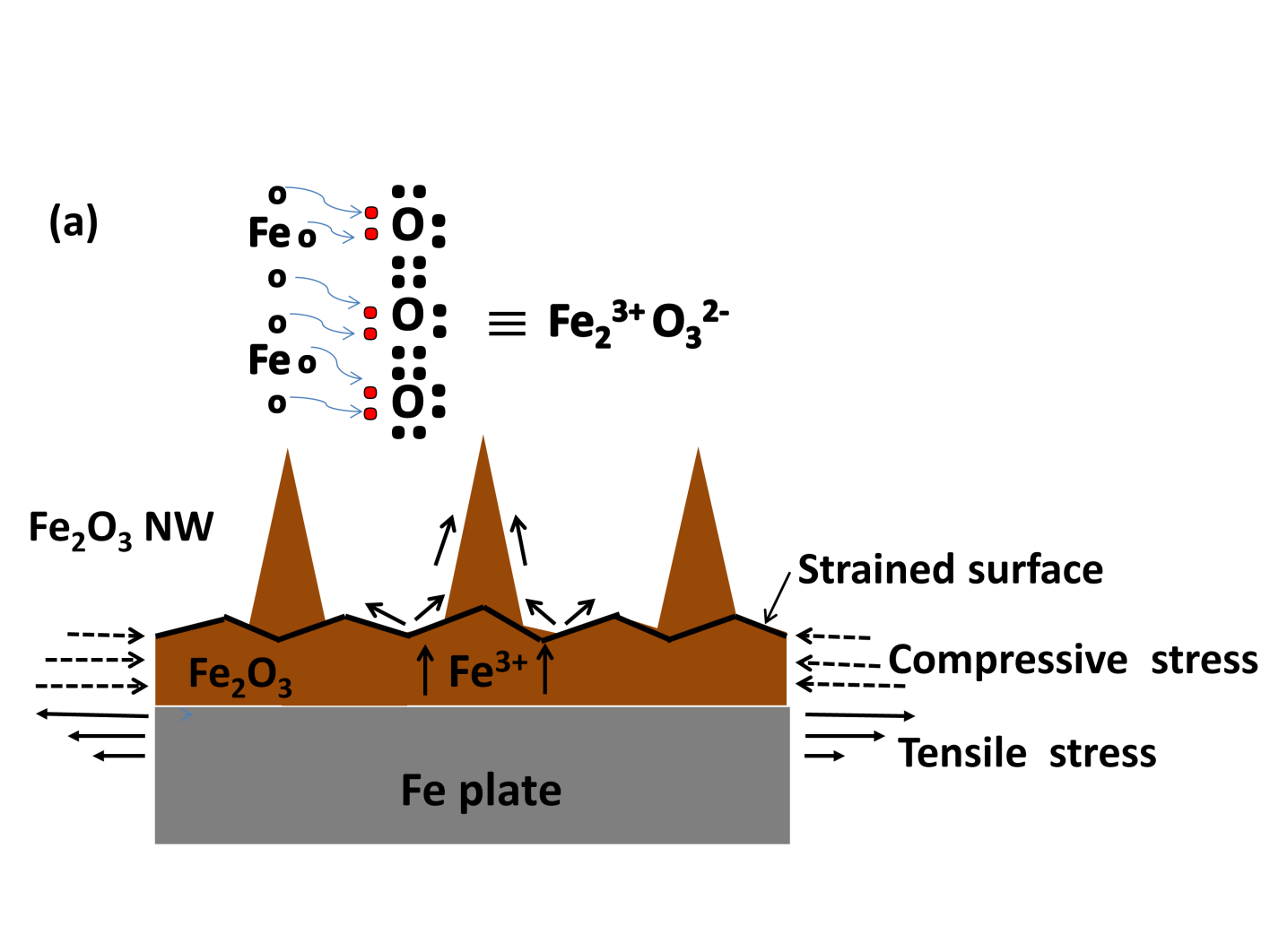


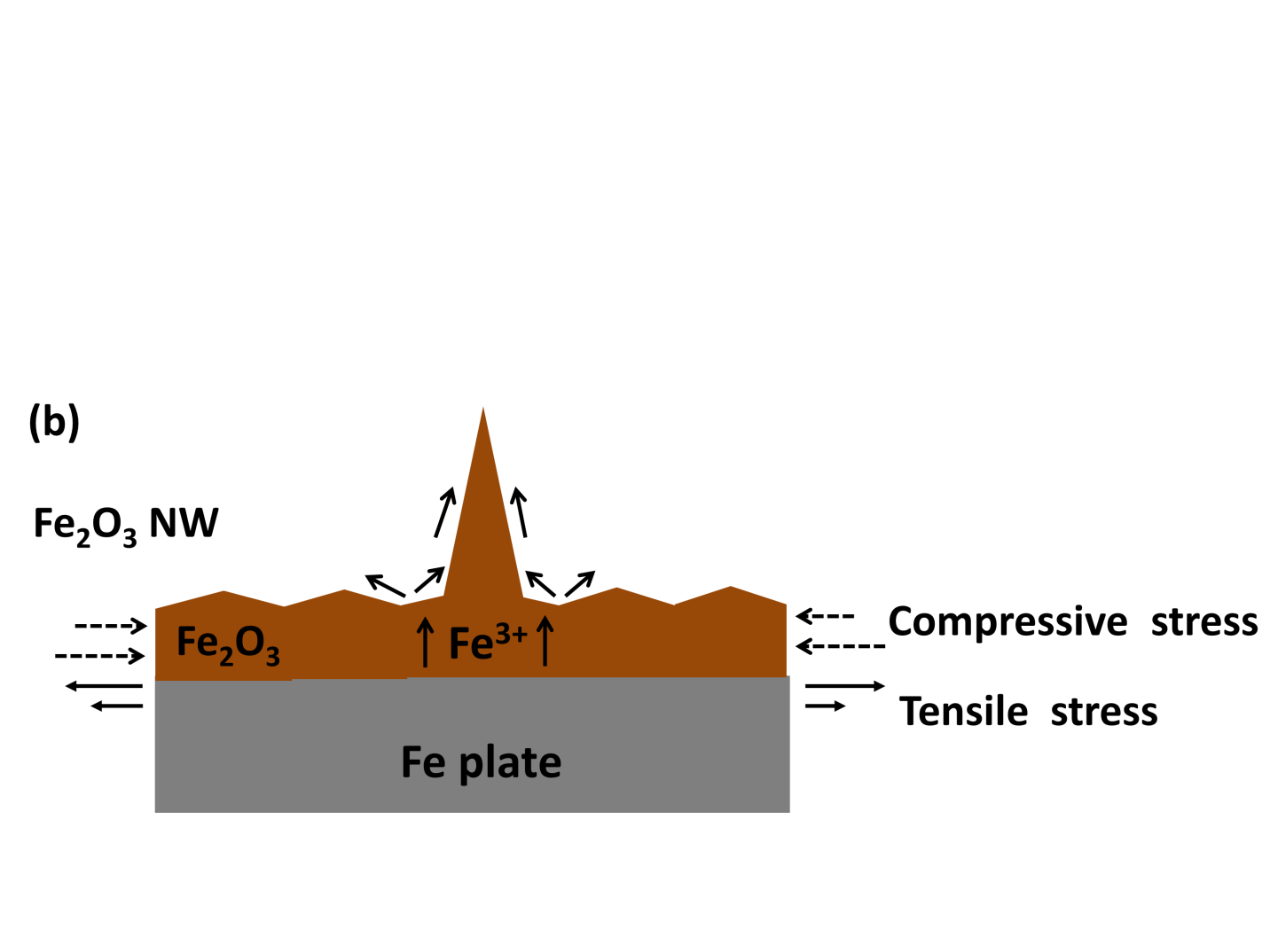


**Figure S2.** Nanowire growth mechanism for (a) surface strained and (b) unstrained Fe plate


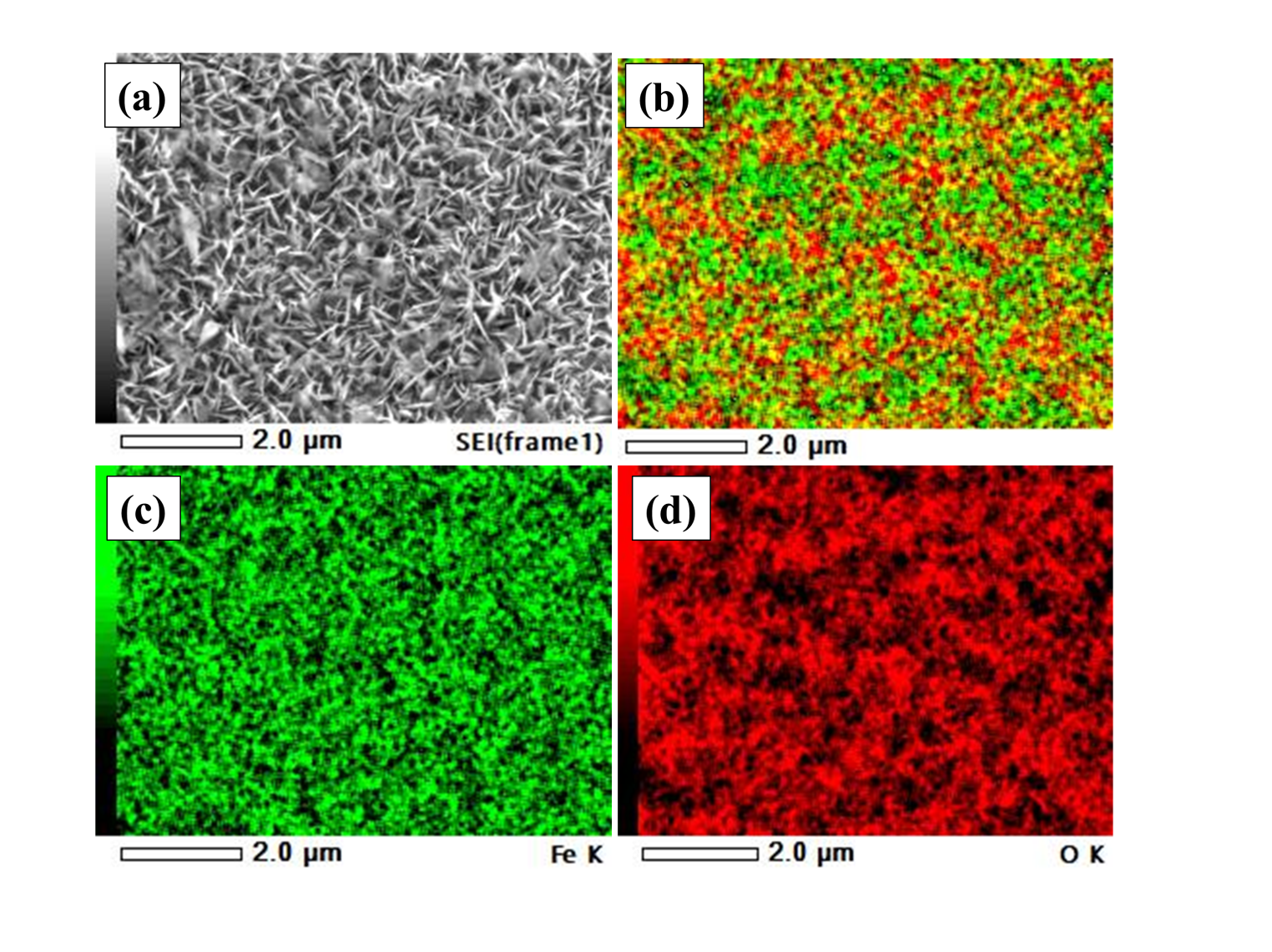


**Figure S3.** (a) FESEM image of Fe_2_O_3_-1 taken from the top view and the corresponding EDS mapping of (b) Fe_2_O_3_, (c) Fe and (d) O


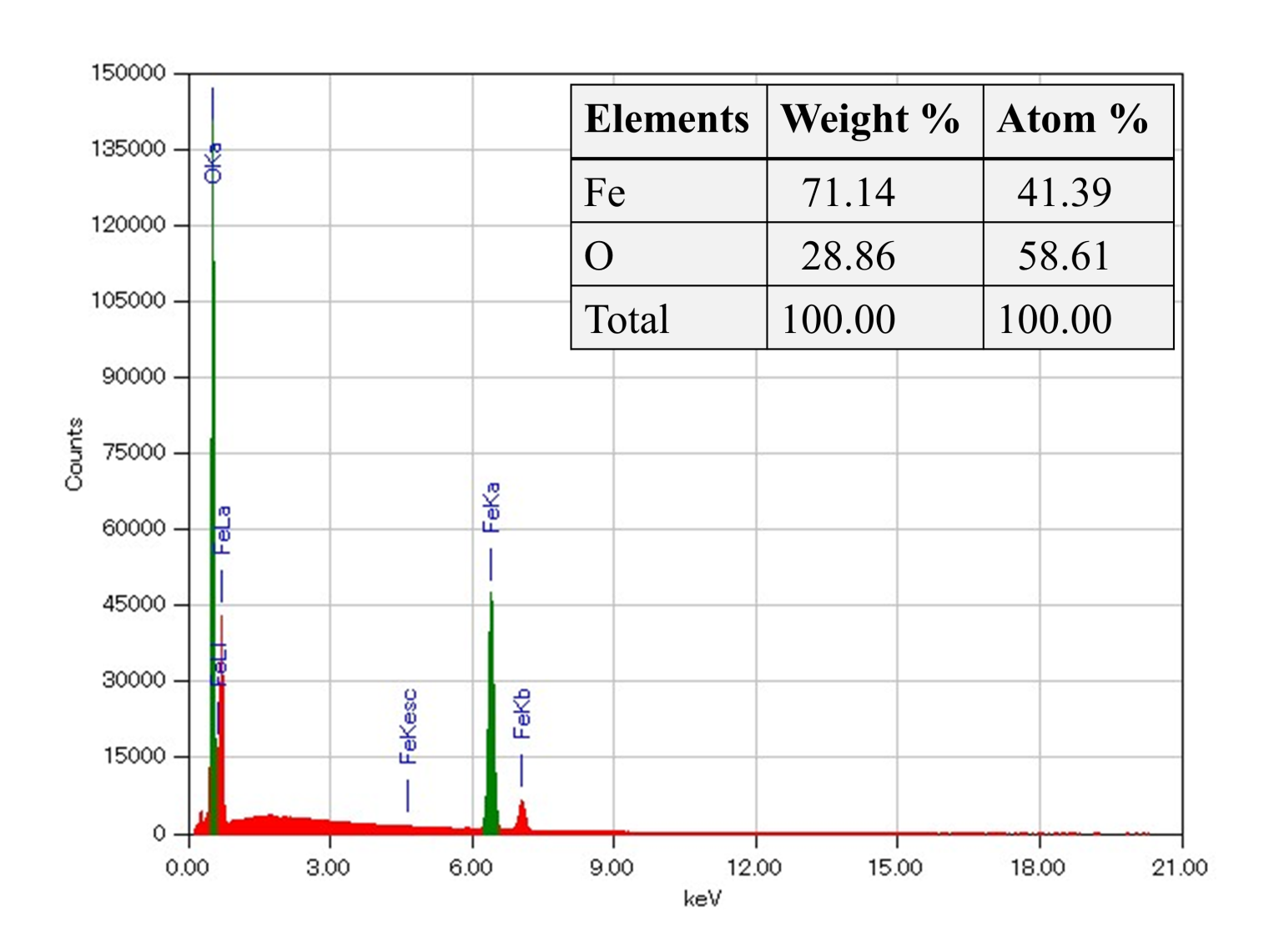


**Figure S4.** EDS spectrum of Fe_2_O_3_-1


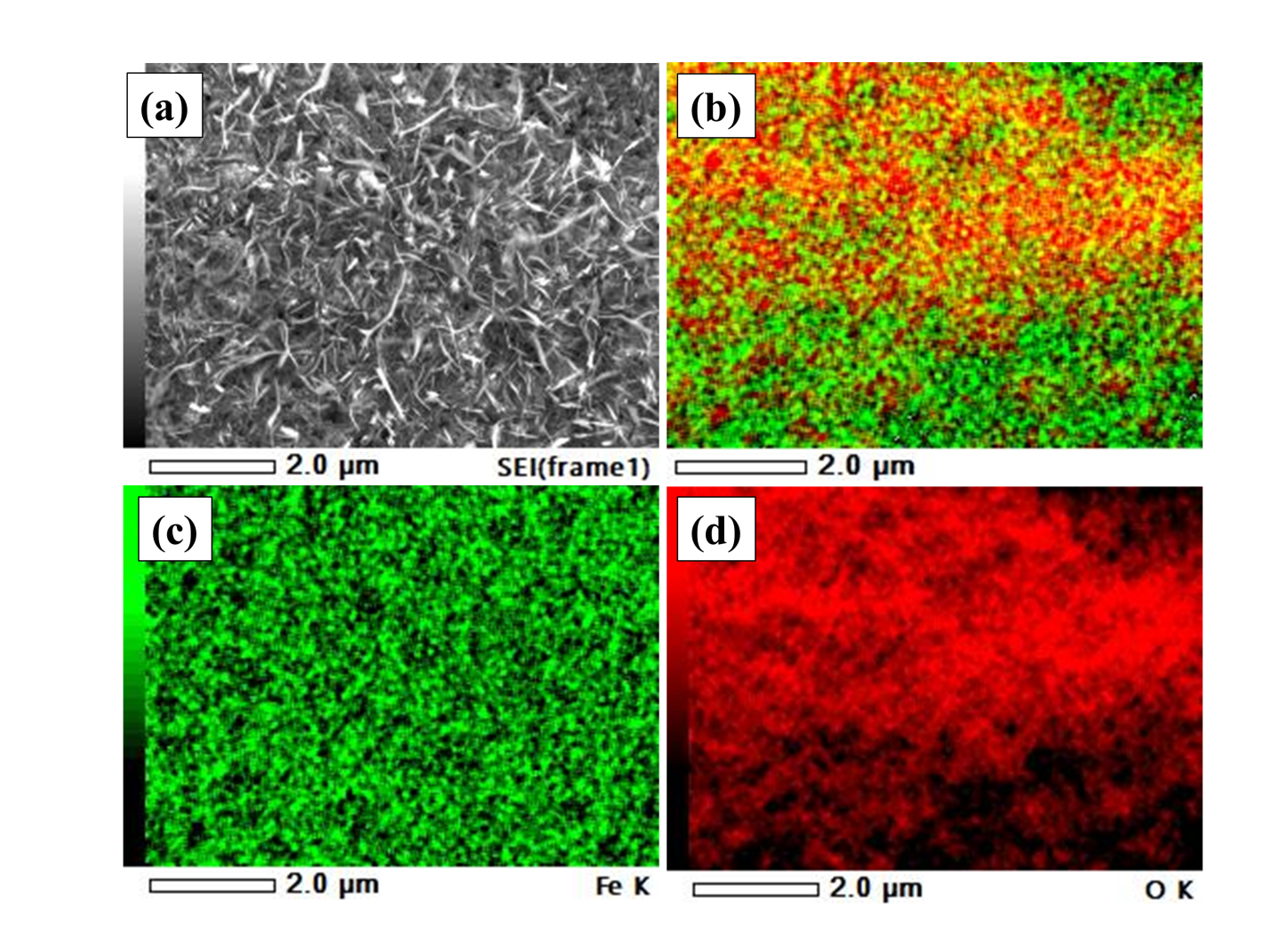


**Figure S5.** (a) FESEM image of Fe_2_O_3_-2 taken from the top view and the corresponding EDS mapping of (b) Fe_2_O_3_, (c) Fe and (d) O


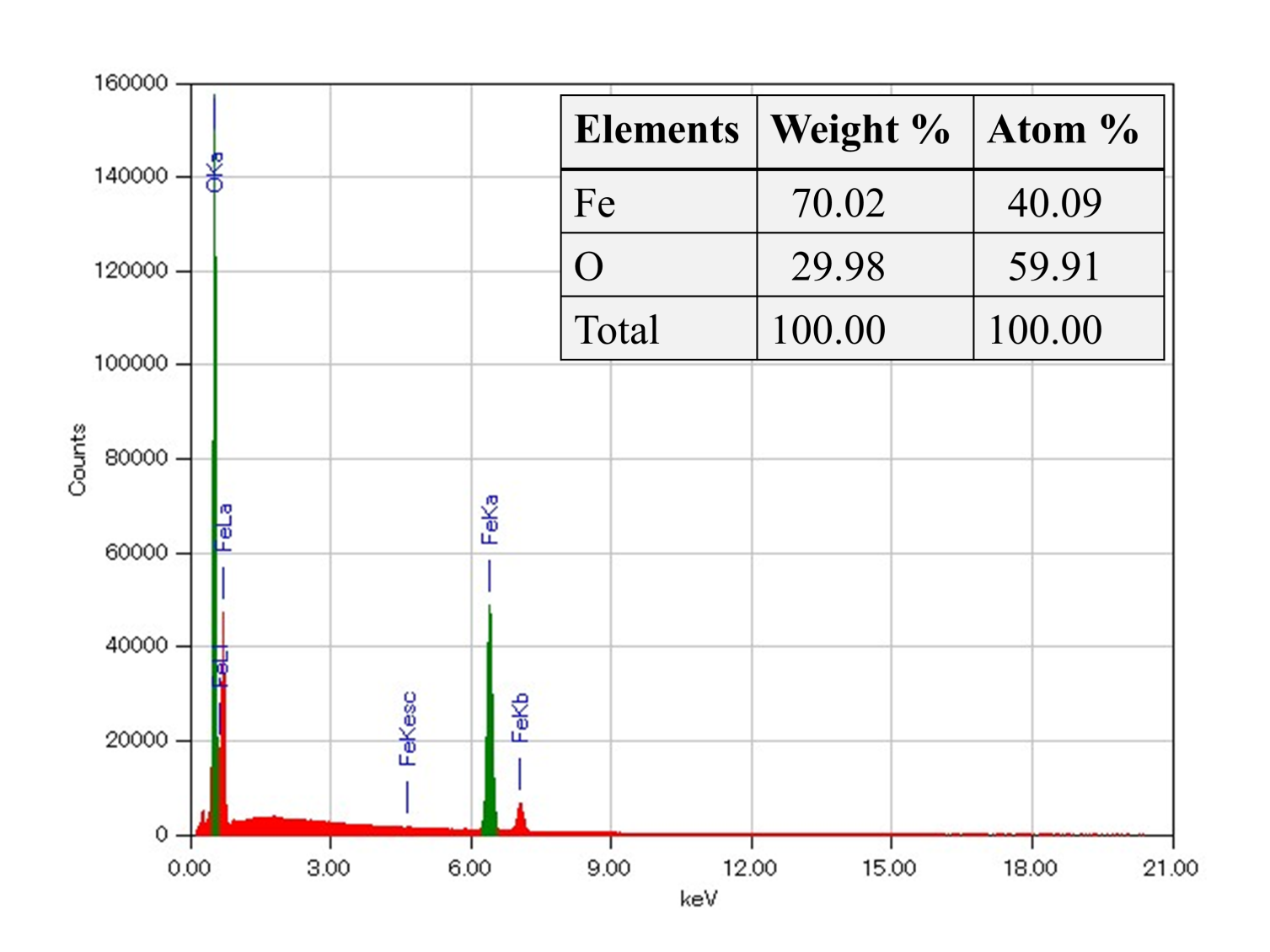


**Figure S6.** EDS spectrum of Fe_2_O_3_-2


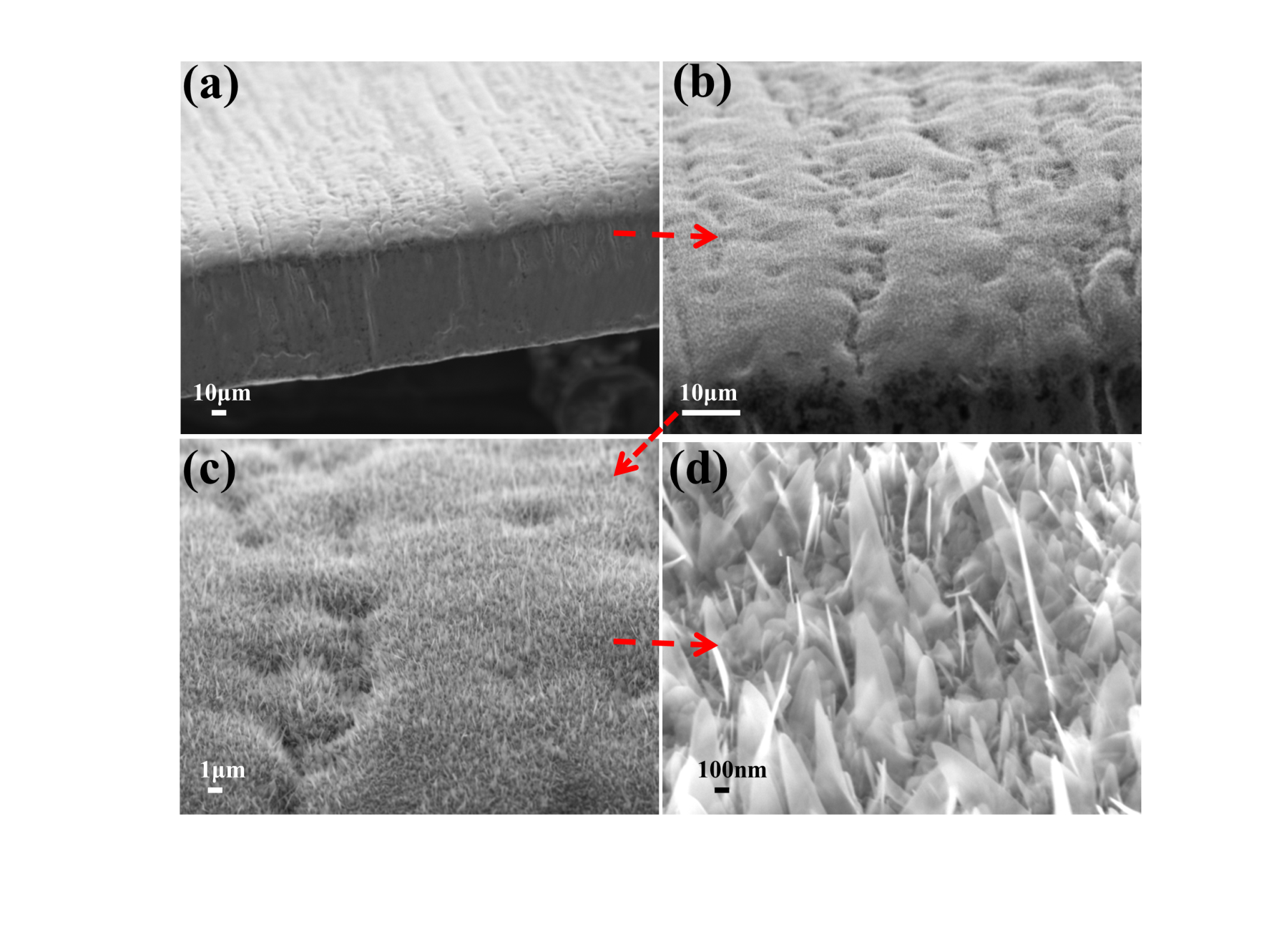


**Figure S7.** Representative FESEM images of Fe_2_O_3_-1 nanowires were captured from low to high magnification (a-d). Images viz., a to d were taken from side view


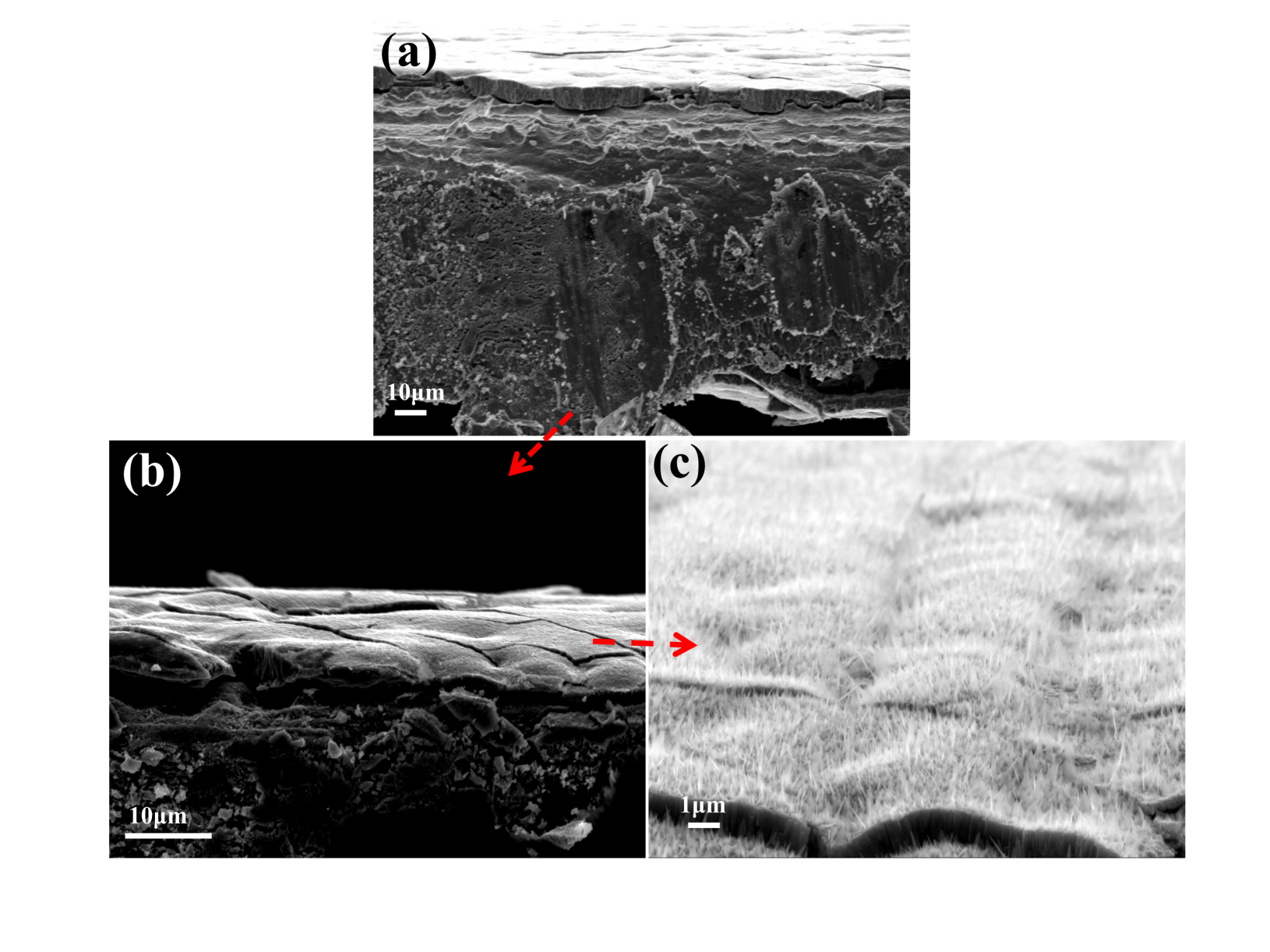


**Figure S8.** Representative FESEM images of Fe_2_O_3_-1 nanowires were captured from low to high magnification (a-c). The cross-sectional images viz., a-c were taken from side view and cracks have occurred on Fe_2_O_3_ layer when cutting the plate with scissors to capture the cross-section image


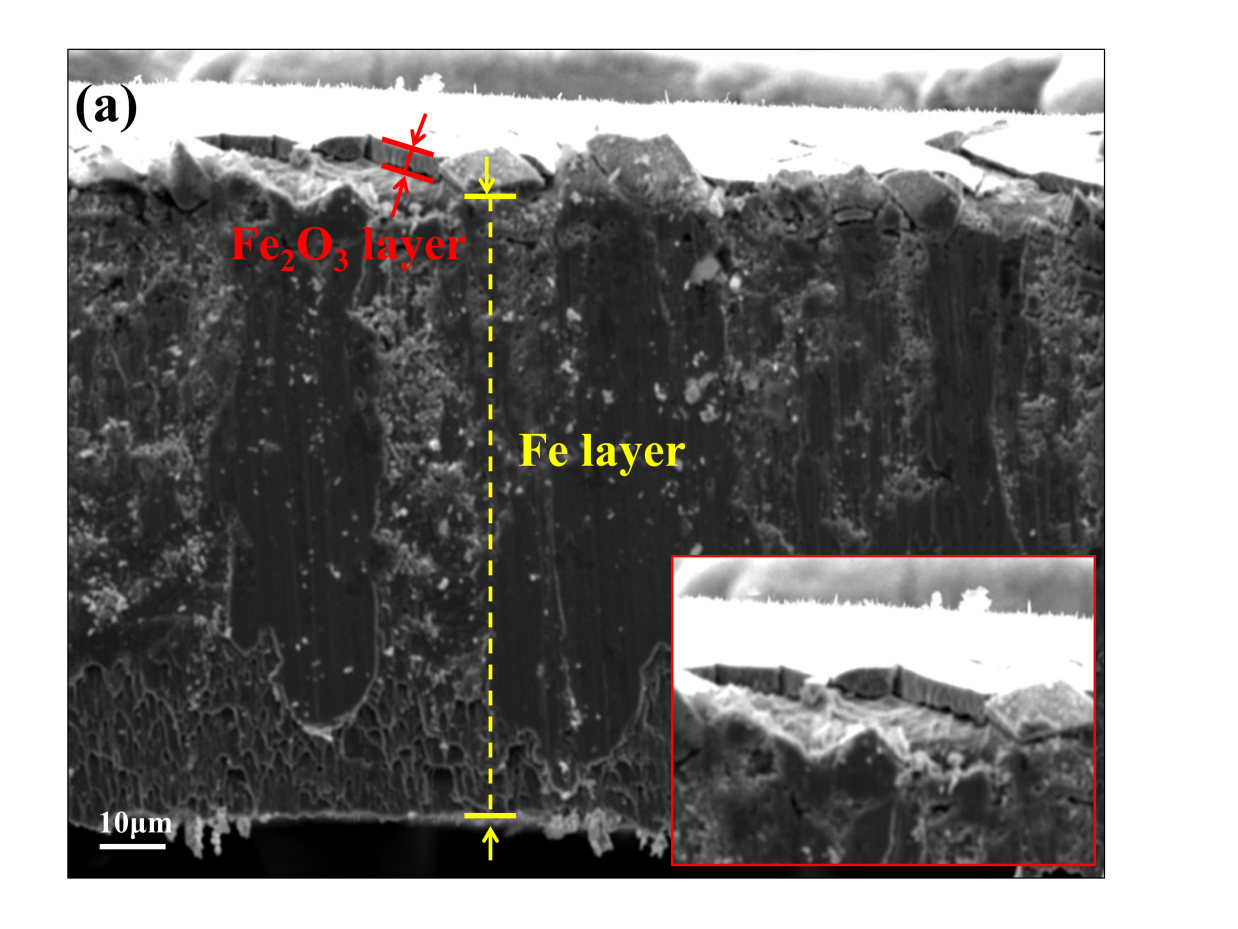


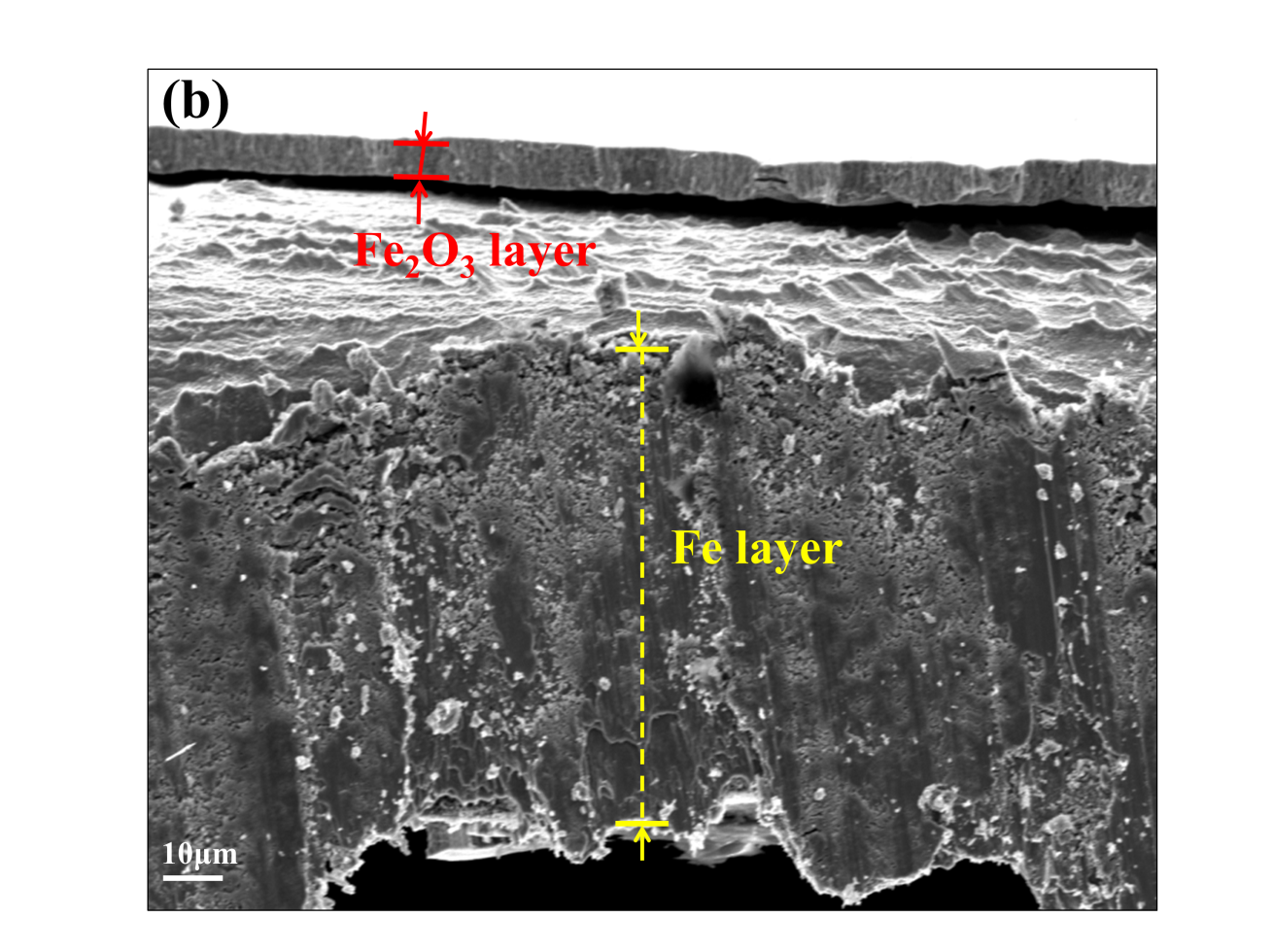


**Figure S9.** Cross-section FESEM images of (a) Fe_2_O_3_-1 (inset shows the enlarged image of Fe_2_O_3_ layer) and (b) Fe_2_O_3_-2. In both samples, Fe_2_O_3_ and Fe layers are differentiated by red and yellow colors, respectively. Average thickness of each layer was measured from different locations. When cutting the sample with scissors, the non-existence of broken parts of Fe_2_O_3_ layer creates gap between Fe_2_O_3_ and Fe layer ((b) Fe_2_O_3_-2)


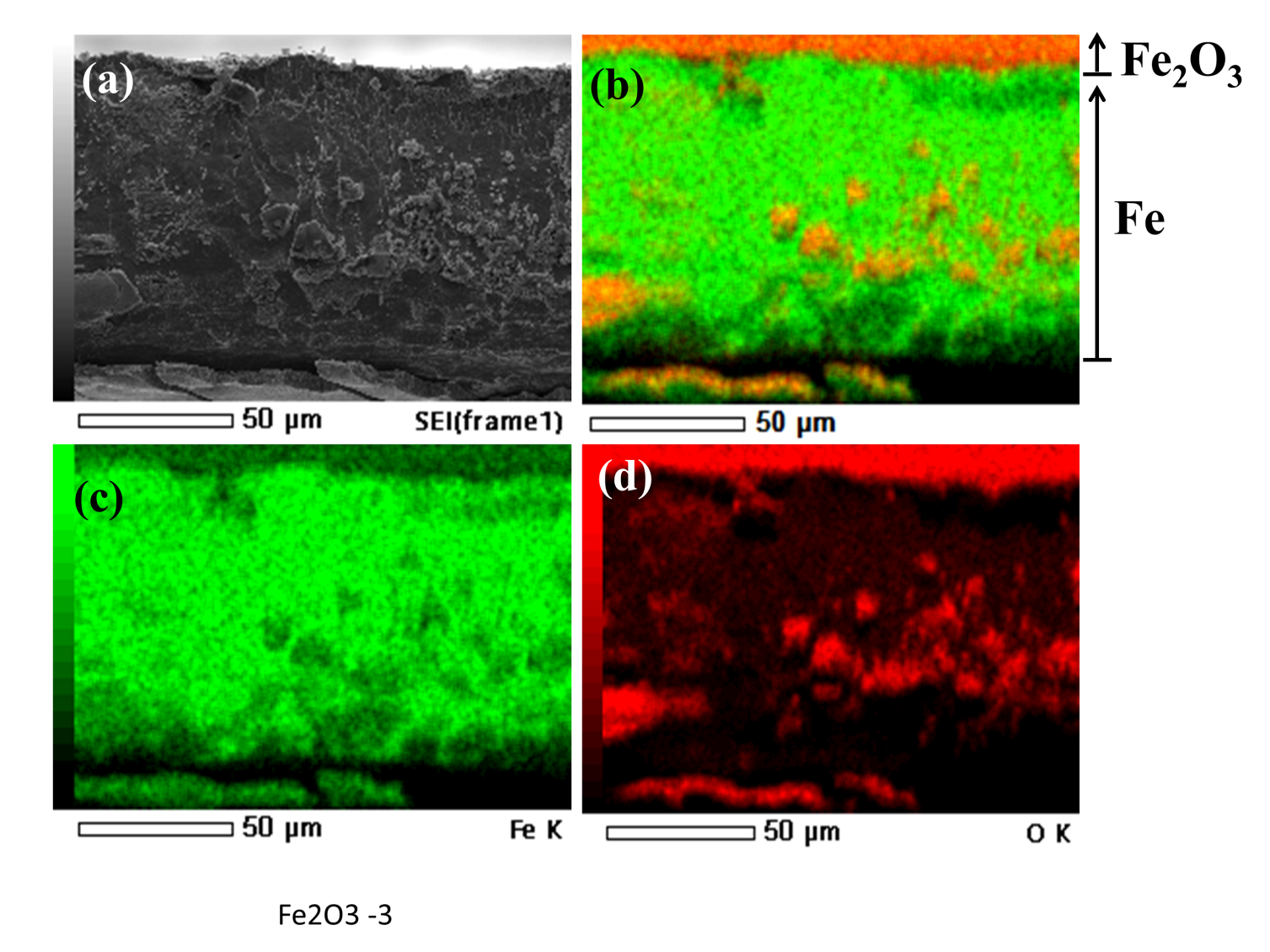


**Figure S10.** (a) Cross-sectional image of Fe_2_O_3_-1, (b) EDS mapping images of Fe_2_O_3_-1 and
(c, d) mapping images of Fe and O in Fe_2_O_3_-1


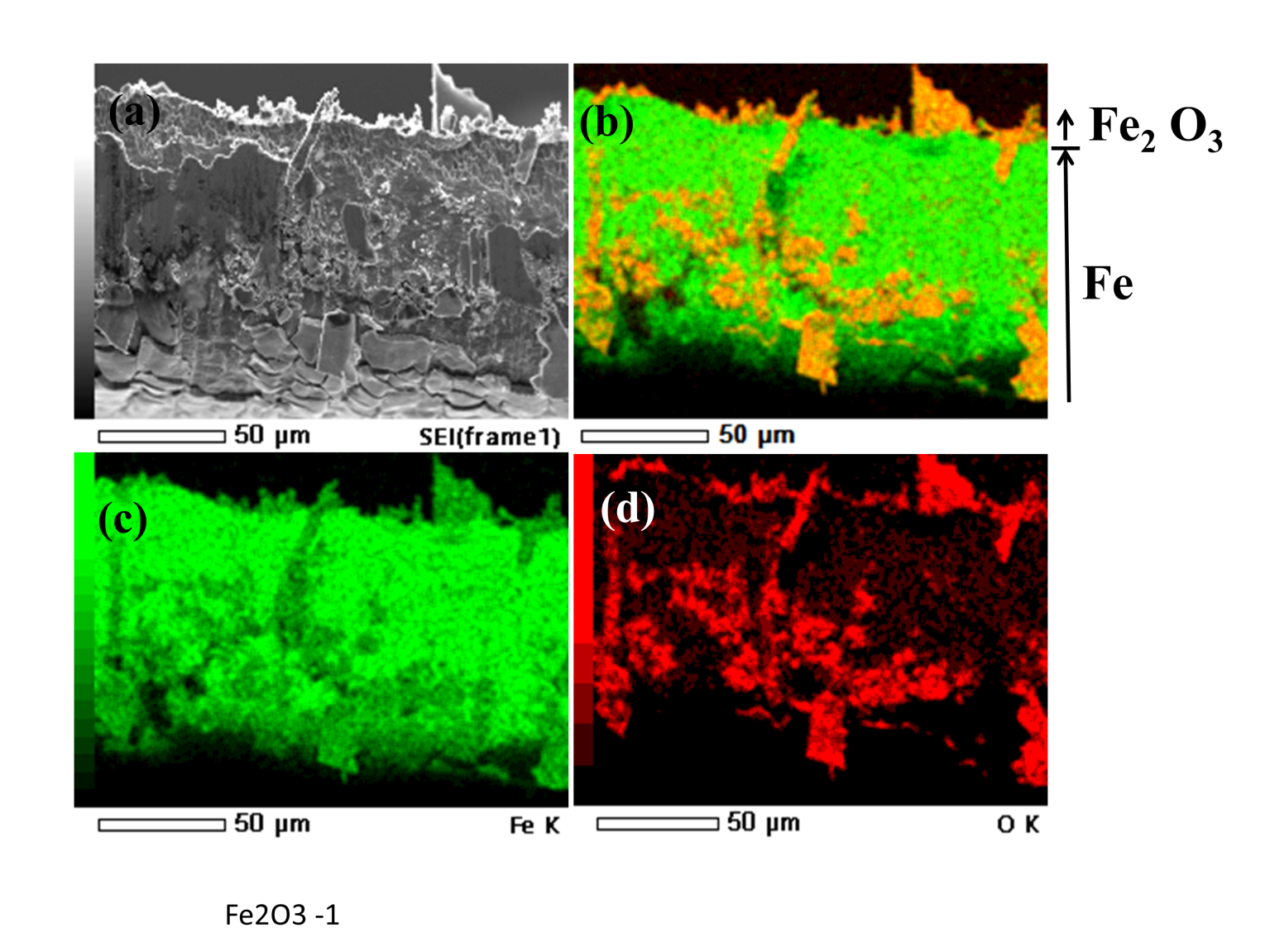


**Figure S11.** (a) Cross-sectional image of Fe_2_O_3_-2, (b) EDS mapping images of Fe_2_O_3_-2 and
(c, d) mapping images of Fe and O in Fe_2_O_3_-2





**Figure S12.** FTIR spectra of (a) Fe_2_O_3_-1 and (b) Fe_2_O_3_-2


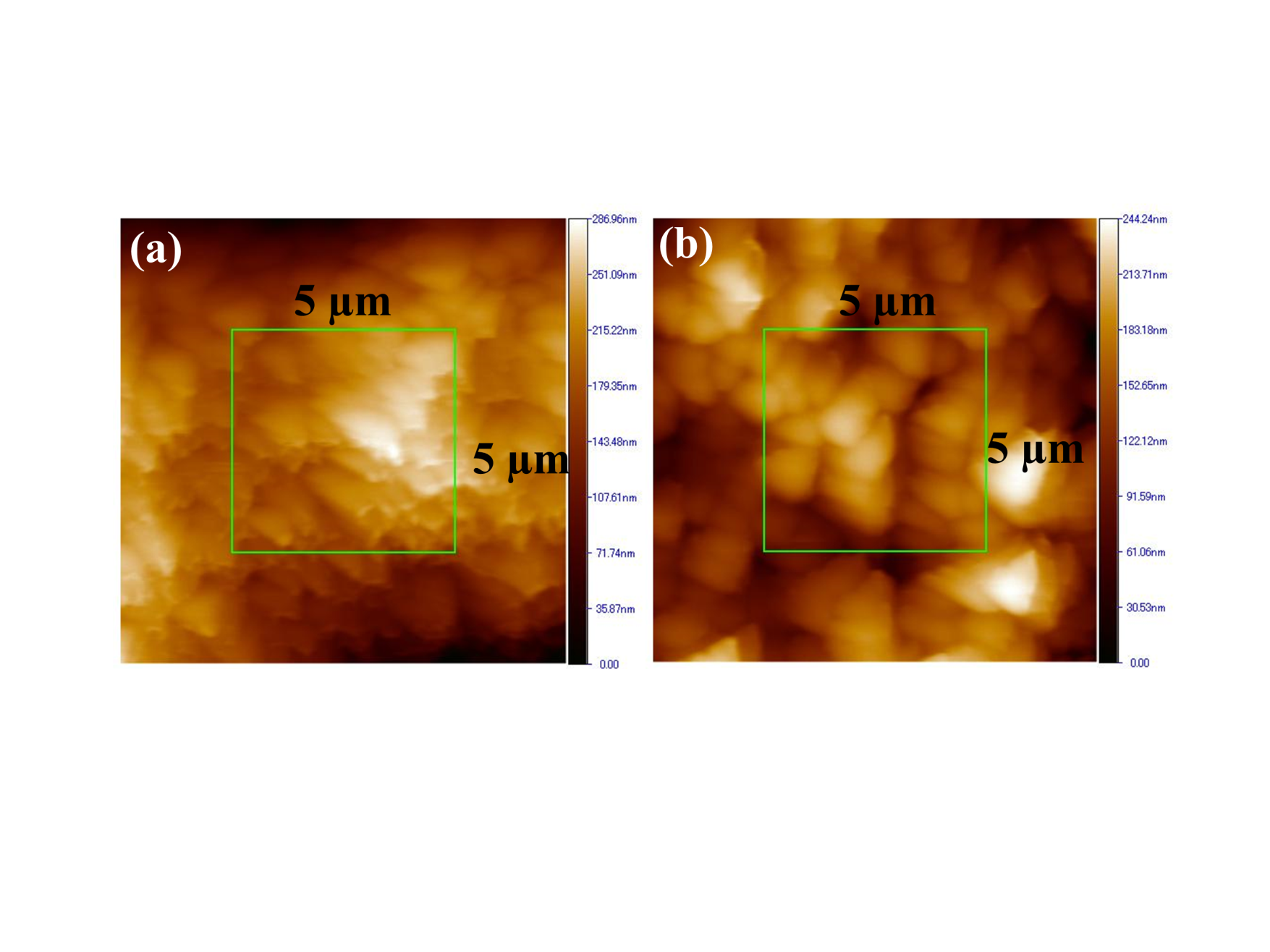


**Figure S13.** Surface topography measurement of the nanowires was performed by Atomic Force Microscopy and 5 x 5 μm area was selected for analysis, and the corresponding roughness and surface area was measured as 42.48 nm and 25.4 μm^2^ for Fe_2_O_3_-1 (a), and 34.86 nm and 25.5 μm^2^ for Fe_2_O_3_-2 (b), respectively





**Figure 14**. LSV of Fe_2_O_3_-1 and Fe_2_O_3_-2 measured after stability test (at 50 mV/sec scan rate in 1M NaOH alkaline solution)


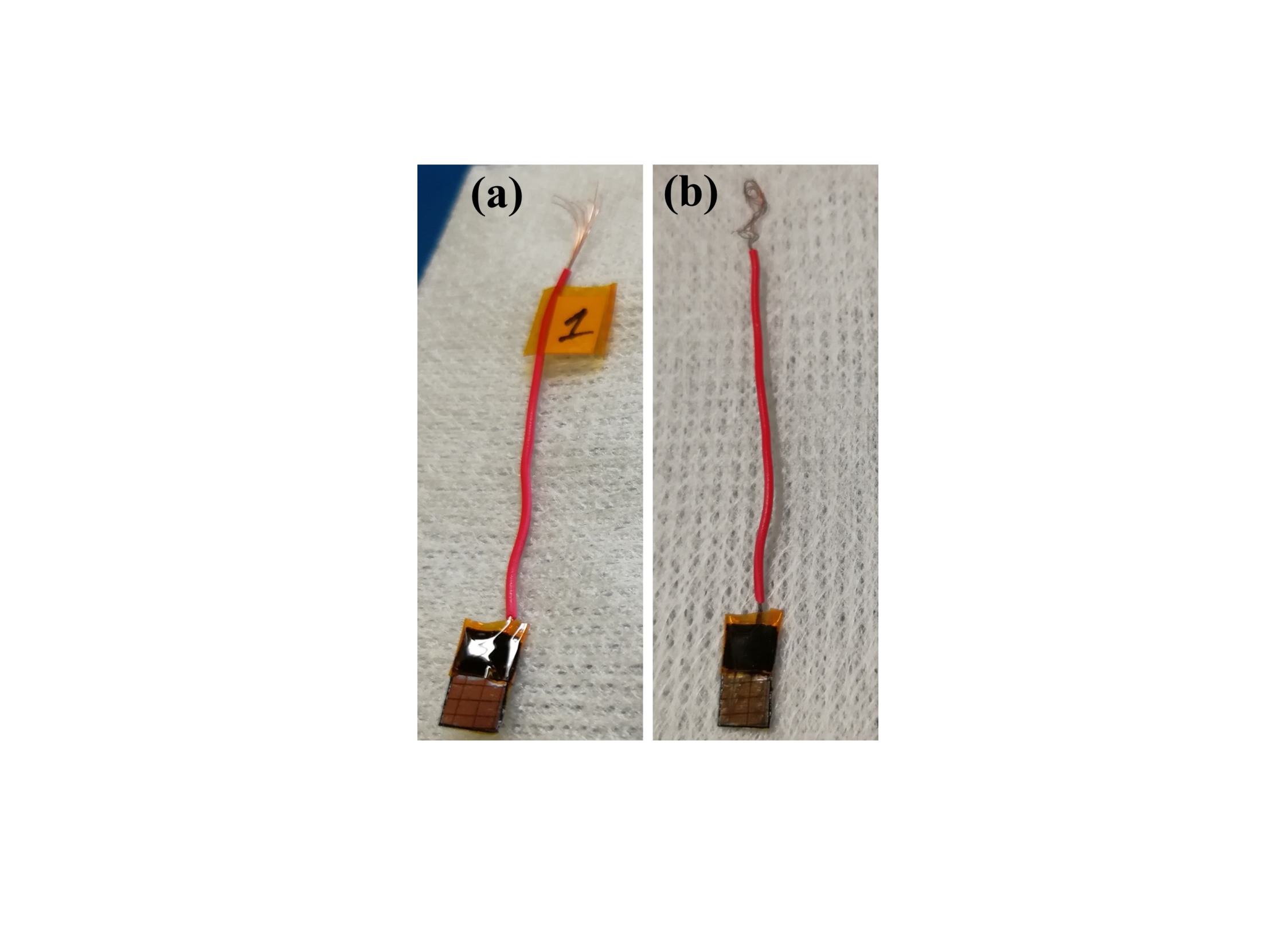


**Figure S15.** Photographs of Fe_2_O_3_-1 electrode captured (a) before and (b) after electrochemical stability measurement


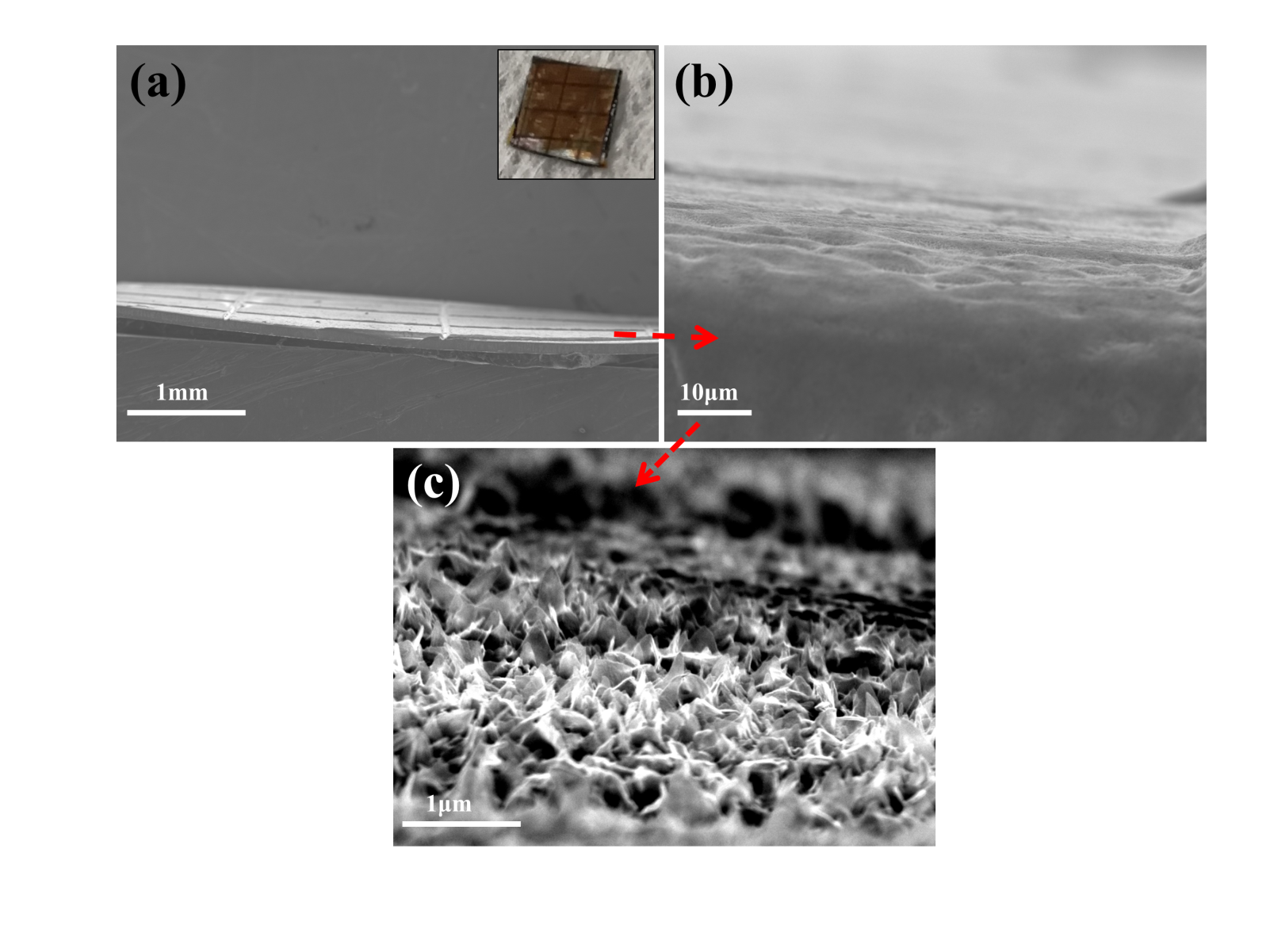


**Figure S16.** Low to high magnification FESEM images of Fe_2_O_3_-1 NWs (a-c) after electrochemical measurement (inset is the photograph of Fe_2_O_3_-1 electrode used for electrochemical measurement)


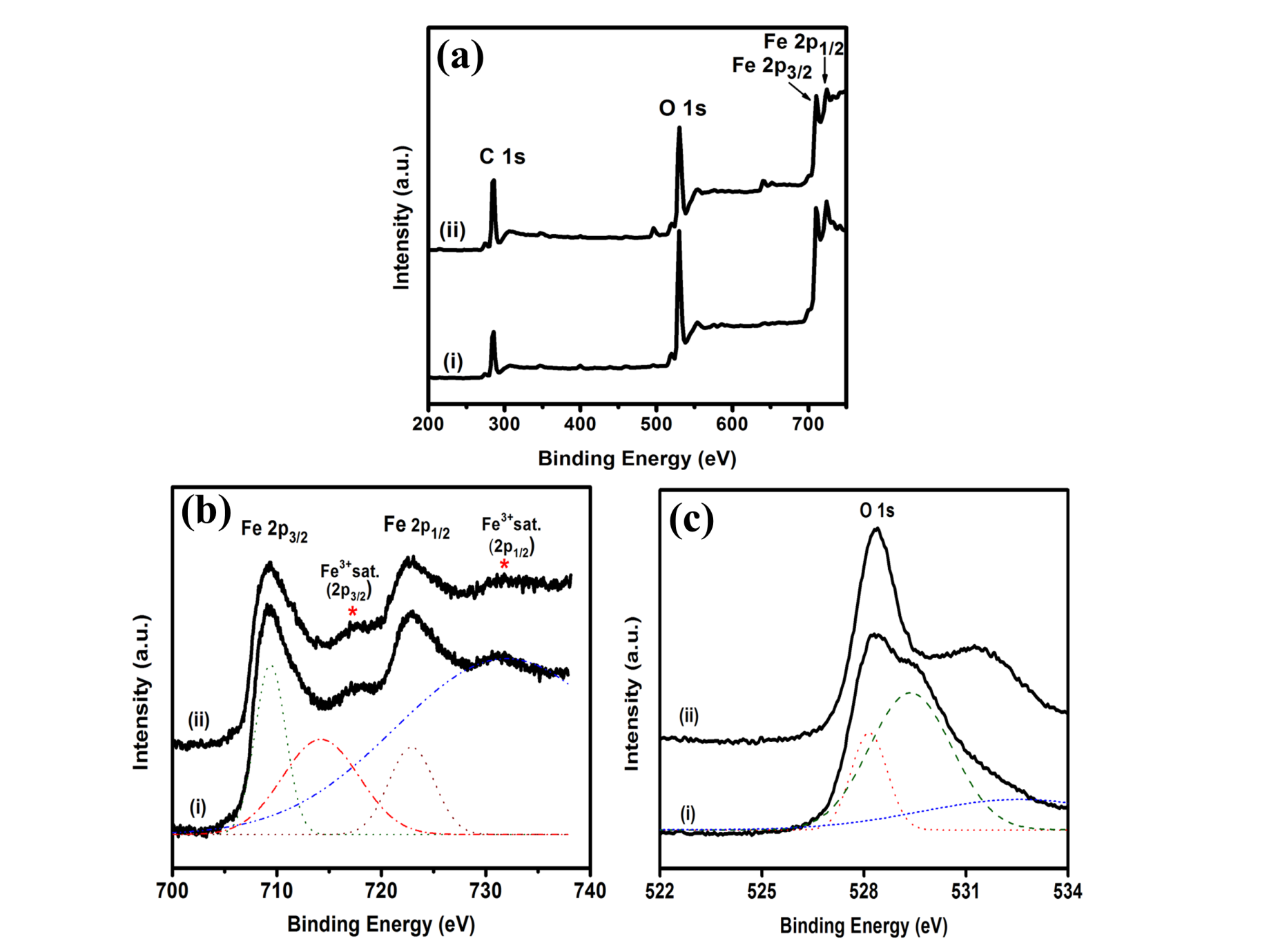


**Figure** **S17.** XPS analysis performed for Fe_2_O_3_-1 and Fe_2_O_3_-2 after stability measurement.
(a) XPS survey spectra of (i) Fe_2_O_3_-1 and (ii) Fe_2_O_3_-2. Deconvoluted XPS spectra of (b) Fe 2p and (c) O 1s of (i) Fe_2_O_3_-1 and (ii) Fe_2_O_3_-2
